# Metabolic and Transcriptional Changes across Osteogenic Differentiation of Mesenchymal Stromal Cells

**DOI:** 10.3390/bioengineering8120208

**Published:** 2021-12-10

**Authors:** Thora Bjorg Sigmarsdottir, Sarah McGarrity, Adrián López García de Lomana, Aristotelis Kotronoulas, Snaevar Sigurdsson, James T. Yurkovich, Ottar Rolfsson, Olafur Eysteinn Sigurjonsson

**Affiliations:** 1 School of Science and Engineering, Reykjavík University, 101 Reykjavik, Iceland; thoras13@ru.is (T.B.S.); sarahm@hi.is (S.M.); 2 Center for Systems Biology, University of Iceland, 101 Reykjavik, Iceland; adrian@hi.is (A.L.G.d.L.); aris@hi.is (A.K.); ottarr@hi.is (O.R.); 3 Biomedical Center, University of Iceland, 101 Reykjavik, Iceland; snaevar@hi.is; 4 Department of Bioengineering, University of California San Diego, La Jolla, CA 92093, USA; jyurkovich@ucsd.edu; 5 The Blood Blank, Department of RnD, Landspitali–The National University Hospital of Iceland, 101 Reykjavik, Iceland

**Keywords:** mesenchymal stromal cells (MSCs), osteogenic differentiation, metabolites, metabolism, metabolic changes, glycolysis, oxidative phosphorylation, gene regulatory network

## Abstract

Mesenchymal stromal cells (MSCs) are multipotent post-natal stem cells with applications in tissue engineering and regenerative medicine. MSCs can differentiate into osteoblasts, chondrocytes, or adipocytes, with functional differences in cells during osteogenesis accompanied by metabolic changes. The temporal dynamics of these metabolic shifts have not yet been fully characterized and are suspected to be important for therapeutic applications such as osteogenesis optimization. Here, our goal was to characterize the metabolic shifts that occur during osteogenesis. We profiled five key extracellular metabolites longitudinally (glucose, lactate, glutamine, glutamate, and ammonia) from MSCs from four donors to classify osteogenic differentiation into three metabolic stages, defined by changes in the uptake and secretion rates of the metabolites in cell culture media. We used a combination of untargeted metabolomic analysis, targeted analysis of ^13^C-glucose labelled intracellular data, and RNA-sequencing data to reconstruct a gene regulatory network and further characterize cellular metabolism. The metabolic stages identified in this proof-of-concept study provide a framework for more detailed investigations aimed at identifying biomarkers of osteogenic differentiation and small molecule interventions to optimize MSC differentiation for clinical applications.

## 1. Introduction

Around 2.2 million surgeries involving bone grafts are performed annually worldwide, a number expected to grow due to a global rise in the geriatric population. This will create an increased demand for graft material, which may pose a challenge because all current sources have limitations ranging from a lack of safety or osteogenic potential to naturally limited supply [1,2,3,4,5,6,7]. To meet the ever-growing need for materials while eliminating the risk to donors, the therapeutic application of mesenchymal stromal cells (MSCs) has been on the rise [6]. MSCs are multipotent, post-natal, self-renewing stem cells that can be isolated from various adult tissue types (e.g., fat, peripheral blood, and bone marrow) and are defined by the ability to differentiate in vitro into cells of mesodermal origin: osteoblasts, chondrocytes, and adipocytes [8]. The progression of differentiation depends on various factors, including hormonal and local factors, forming regulatory and metabolic networks [9].

Osteogenic differentiation occurs in three stages. First, the proliferation of osteoprogenitor cells is promoted by various growth factors, including insulin-like growth factor (IGF), transforming growth factor (TGF), and fibroblast growth factor (FGF). Next, differentiation into osteoblasts and the production of the extracellular matrix takes place, promoted by both hormonal and growth factors, many of which act by increasing the activity of Runt-related transcription factor 2 (RUNX2) and its downstream genes. Finally, the progressive mineralization of bone matrix proteins within the extracellular matrix creates bone tissue that is capable of withstanding stress and strain [9].

Currently, we have an incomplete understanding of cellular metabolism during osteogenic differentiation—most of which is based on extracellular metabolite studies or the mapping of enzyme activity—based on in vitro differentiated MSCs [10,11,12,13]. During proliferation, MSCs preferentially use anaerobic glycolysis for energy production, as indicated by low mitochondrial activity [11]. During osteogenic differentiation, mitochondrial biogenesis increases with a shift into aerobic energy production through oxidative phosphorylation [12,14]. The metabolic shift from reliance on glycolysis to the activation of oxidative phosphorylation is important because it makes osteogenically differentiated MSCs more dependent upon oxygen; a reliance on oxygen may explain the reduced osteogenic differentiation under hypoxic conditions. Additionally, the activation of glutamine anaplerosis has been reported for cells in osteogenic differentiation, further indicating a greater reliance on mitochondrial energy production [15,16,17]. However, we lack a mechanistic understanding of the underlying metabolic processes that govern osteogenic differentiation, which represents a barrier to the development of successful clinical therapeutics [6,18]. The quantitative characterization of metabolic and gene expression regulatory changes across this process can serve as a foundational resource toward a better understanding of osteogenic differentiation.

In this study, we initially quantified changes in metabolite biomarkers for cellular energy production. We observed three distinct metabolic stages during osteogenic differentiation, as defined by the temporal changes observed in the media concentrations of the five key metabolites that accompany MSC osteogenesis. Next, we performed the untargeted analysis of intracellular metabolomic data as well as the targeted analysis of ^13^C-glucose-labeled intracellular metabolomic data to identify which pathways were contributing to the observed functional differences. Furthermore, to elucidate the mechanistic underpinnings of these stages, we built a gene regulatory network from the quantified gene expression changes. This approach resulted in the mechanistic inference of transcription factors (TFs) regulating the set of differentially expressed genes (DEGs). Taken together, this proof-of-concept study provides a molecular basis for the observed metabolic phenotypic shifts that may serve as a foundation for future, more in-depth studies into the optimization of MSC osteogenic differentiation.

## 2. Materials and Methods

### 2.1. Cell Culture and Osteogenic Differentiation

Human bone marrow-derived MSCs from four donors were purchased from Lonza (Basel, Switzerland). All experiments were performed in triplicate and cells were cultured under normoxic conditions at 5% CO_2_, 37 °C, and 95% humidity. Cells were maintained in basal growth medium (BGM). BGM was prepared by adding 5000 IU/mL of heparin (LEO Pharma A/Sm, Ballerup, Denmark), 1% penicillin/streptomycin (Gibco, Grand Island, NE, USA), and 10% pathogen inactivated platelet lysate (PIPL) supernatant (Platome, Reykjavik, Iceland) into DMEM/F12 + Glutamax growth medium (Gibco, Grand Island, NE, USA). The PIPL was centrifuged at 5000 rpm (4975× *g*) for 10 min before the resulting supernatant was added to the medium.

Cells were used for osteogenic differentiation experiments at passages 2, 3, and 5. Osteogenic differentiation was initiated using osteogenic culture medium (OM), prepared from 45 mL of BGM supplemented with dexamethasone (50 μL of 0.1 mM stock solution, Sigma, St. Louis, MO, USA), Bone Morphogenetic Protein 2 (BMP-2, 50 μL of 50 ng/µL stock solution, Peprotech, Rocky Hill, CT, USA), β-glycerophosphate (108 mg, Sigma), and ascorbic acid (50 μL of 50 mM stock solution, Sigma).

For the collection of samples for metabolite analyses (glucose, lactate, glutamine, glutamic acid, and ammonia), cells were seeded at 5000 cells/cm^2^ in 25 cm^2^ culture flasks with 5 mL OM. Medium was changed approximately every 48 h and samples of spent medium (0.5 mL) were taken in duplicate from each of the culture flasks approximately every 24 and 36 h for cells under osteogenic differentiation. All samples were stored at −80 °C until analysis.

### 2.2. Alkaline Phosphatase Assay

The enzymatic activity of ALP during osteogenic differentiation was measured for all four donors using standard procedures. Cells were grown for 7, 14, or 28 days in 12-well plates at 3500 cells/cm^2^ with 0.75 mL OM. For the Day 0 (control) samples, cells were collected from BGM prior to culturing. After the relevant culture period, cells were washed with phosphate-buffered saline (PBS) and then lysed for protein extraction with 0.02% Triton-100 (Sigma) in PBS. A liquid p-nitrophenyl phosphate solution was then added at a 1:1 ratio and incubated at 37 °C for 30 min in the dark before being measured using a Multiskan Spectrum spectrometer (Thermo Scientific, Helsinki, Finland). The ALP activity in terms of the conversion of p-nitrophenyl phosphate into p-nitrophenol was calculated using Equation (1):

(1) $$ALP\;activity\;\left[\frac{\mathrm{nmol}}{\min}\right]=\frac{optical\;density}{18.8\;\left[{µ\mathrm{mol}}^{-1}\;\right]\times time\;\left[\min\right]}\times 1000\left[\frac{\mathrm{nmol}}{µ\mathrm{mol}}\right]\;$$

### 2.3. Alizarin Red Staining

Mineralization during osteogenic differentiation was determined by staining using Alizarin Red S indicator (Sigma-Aldrich) according to standard procedures. Cells for Alizarin Red staining were seeded at 3500 cells/cm^2^ in a 12-well plate and grown for up to 28 days with 0.75 mL OM, with the Day 0 samples cultured in BGM. After sampling, cells were washed with PBS and incubated for 15 min with paraformaldehyde. They were then washed again with deionized water before 2% Alizarin Red S solution (pH 4.1–4.3) was added. The samples were stained for 20 min on a rotating shaker then washed with deionized water and dried overnight. After drying, the samples were rehydrated by adding 1 mL of deionized water and rehydrated overnight. After rehydration, 1 mL of 10% cetylpyridinium chloride solution was added to the cells and incubated for 15 min at 37 °C on a rotating shaker. The cell layer was then scraped, centrifuged for 10 min at 16,100× *g* and 24 °C, and the resulting supernatant was measured for optical density in a Multiskan Spectrum spectrometer (Thermo Scientific) at 562 nm.

### 2.4. Gene Expression/q-PCR Analysis

After culturing for 28 days, cells were harvested with trypsin and TRI reagent (Ambion, Austin, TX, USA). All samples were kept in RNase-free microcentrifuge tubes at −80 °C until RNA isolation was performed. To perform the isolation, 150 μL of chloroform (Merck, Darmstadt, Germany) was added to all samples, after which they were centrifuged for 10 s at 12,000× *g* and 4 °C and the resulting supernatant was transferred to an RNase-free elution tube. Then, 500 μL of isopropanol (Merck) was added, samples were centrifuged for 8 min at 12,000× *g* and 10 °C, and the resulting supernatant was discarded. Then 1 mL of 75% ethanol was added, and samples were centrifuged for 5 min at 7500× *g* and 22 °C. The RNA pellet was air dried before 50 μL of RNase-free water (Qiagen, Hilden, Germany) was added and the solution incubated at 58 °C on a PHMT Thermoshaker heat block (Grant Instruments, Shepreth, UK) for 12 min. Then, 300 μL of RLT buffer (Qiagen) with 0.01% β-mercaptoethanol (Sigma) and 350 μL of 70% ethanol (Gamla Apótekið, Reykjavik, Iceland) were added and the samples were transferred to an RNeasy spin column. The columns were centrifuged for 15 s at 16,100× *g* and 24 °C. Next, 700 μL of RW1 buffer (Qiagen) was added and the columns centrifuged again for 15 s at 16,100× *g* and 24 °C and then 500 μL of RPE buffer (Qiagen) was added to the columns and they were centrifuged again. After a final centrifugation step using the same settings, the column was placed in a new 1.5-mL collection tube and 50 μL of RNase-free water (Qiagen) was added to elute the RNA. The resulting flow-through samples were then stored at −80 °C until cDNA synthesis was performed.

For cDNA synthesis, isolated RNA samples were mixed in a new Ranse-free cDNA tube (Nunc, Roskilde, Denmark) at a 1:1 ratio with a master mix (8 μL nuclease-free H_2_O, 5 μL 10X RT buffer, 5 μL 10X RT random primers, 2.5 μL RNase inhibitor, 2 μL 25X dNTP mix, and 2.5 μL MultiScribe^TM^ Reverse transcriptase per sample) prepared from a High-Capacity cDNA Reverse Transcription Kit (Applied Biosystems, Foster City, CA, USA) and nuclease-free H_2_O (Qiagen). All samples and master mix components were thawed on ice prior to use. The mixed samples were centrifuged using a 96-well centrifuge adapter for a few seconds at 2500 rpm before being put into a thermal cycler (Applied Biosystems) operated under the following conditions: 27 °C for 10 min; 37 °C for 120 min; 85 °C for 5 s; 4 °C until use; storage at −20 °C.

Real-time qPCR analysis of the prepared cDNA was then performed, with *RUNX2* and *COL1A2* assayed as the genes of interest. TATA box binding protein (TBP) was used as a housekeeping gene. 10X random primers were used (Applied Biosystems). All samples were mixed using an assay solution of 1 μL Taqman Assay and 10 μL Master Mix plus 2 µL cDNA. All *RUNX2* primers and probes were obtained from Integrated DNA Technologies as premixed assays but *COL1A2* primers/probes were obtained separately from Integrated DNA Technologies. All relevant primer pairs can be found in Supplementary Table S1 online.

### 2.5. Cell Culture and RNA Isolation for RNA-Sequencing

Bone marrow-derived human mesenchymal stem cells from three donors (Lonza, Basel, Switzerland) were taken from liquid nitrogen storage (−180 °C) and seeded into 175 cm^2^ culture flasks (Nunc, Penfield, NY, USA). Approximately 500,000 cells from each donor were used. The media used during the proliferation phase was BGM (45 mL), and media change was performed every 48 h.

Upon reaching approximately 85% confluency, cells were harvested via trypsinization. Media was discarded from the culture flasks, 11.72 mL PBS (Gibco, Grand Island, NE, USA) was used to remove remaining media before 11.72 mL of 0.25% Trypsin (Gibco, Grand Island, NE, USA) was added. Culture flasks were incubated at 37 °C (5% CO_2_, 95% H_2_O) for 5 min before 11.72 mL of BGM was added to neutralize and stop the trypsinization. Both BGM and 0.25% trypsin solution were warmed to 37 °C before use.

The cell solution was transferred to 15 mL falcon tubes and centrifuged at 1750 rpm (609× *g*) for 5 min. The resulting supernatant was discarded and the cells resuspended in 5 mL of BGM. Cells were then counted using a Neubauer hemocytometer (Assistant, Munich, Germany). For osteogenic differentiation of the cells OM (2 mL/well) was used. Cells were seeded on to 6-well plates (200,000 cells/well). Five separate wells were used for each donor in question, one per time point that was to be assessed (Day 3, 6, 9, 16, 28). A total of 15 wells were used. Media was changed every 48 h. Upon the day of assessment, the medium was discarded using a pipette and 1 mL of RNAzol^®^ RT (Molecular Research Center, Inc., Cincinnati, OH, USA) was added to the wells in question in order to lyse the cells. Repeated pipetting was used to dislodge and lyse the cells completely before moving the resulting lysate to a 2-mL Eppendorf tube. In order to precipitate DNA, protein and polysaccharides 0.4 mL of water was added to the lysate, the mixture shaken for 15 s and then stored at room temperature (RT) for 15 min. Samples were then centrifuged for 15 min at 12,000× *g*. Approximately 75% of the resulting supernatant was moved to a new Eppendorf tube. In order to precipitate the total RNA, 1 mL of isopropanol was added to the supernatant collected in the previous step. Samples were then incubated at RT for 10 min before being centrifuged for 10 min at 12,000× *g*. This collected a small RNA pellet at the bottom of the Eppendorf tube. The supernatant was discarded, and the resulting pellet washed twice by adding 0.5 mL 75% ethanol and centrifuging for two minutes at 5000× *g*. The pellet was then dissolved in 25 µL of RNase-free water to gain 20 ng/µL concentration for RNA sequencing.

### 2.6. Cell Culture and Sample Processing for Intracellular Metabolomics Data

Cell cultures (*n* = 3) used to gather unlabeled intracellular metabolomics data were grown in 6-well plate culture vessels (15, 600 cells/cm^2^). Five separate wells were used for cells from each donor to match the desired day of culture (selected based on change point analysis performed on extracellular metabolite data). Upon seeding, the cells were immediately placed in unlabeled OM, with media change (1.5 mL) occurring every 48 h until sample collection took place.

Upon sample collection, all media was discarded. Cells were then washed 3 times using 1 mL of PBS.

Next, 1 mL of 80% methanol solution (stored until needed at −20 °C) was pipetted straight into the cells and a cell scraper was subsequently used to dislodge and scrape cells from the bottom of the culture vessel. The resulting methanol-cell suspension was placed in an Eppendorf tube and stored at −80 °C until needed for metabolite extraction/preparation for mass spectrometry.

### 2.7. Glucose and Lactate Measurements

Extracellular glucose and lactate concentrations were determined in spent medium samples, collected every 24 to 36 h as described above for metabolites, using an ABL90 FLEX blood gas analyzer (Radiometer Medical ApS, Copenhagen, Denmark). Unused medium samples (blank medium) served as the controls.

### 2.8. Glutamine, Glutamic Acid, and Ammonia Measurements

Glutamine, glutamic acid, and ammonia were assayed in the spent medium samples, collected every 24 to 36 h as described for metabolites, using colorimetric assays (Megazyme, Wicklow, Ireland). l-glutamine and ammonia were measured using a single assay (Megazyme) as per the manufacturer’s instructions via determining the decrease in absorbance of the reaction mixture at 340 nm using a Spectromax M3 plate reader (Molecular Devices, San Jose, CA, USA). l-glutamic acid was measured with a separate assay (Megazyme) as per the manufacturer’s instructions via absorbance measurements at 492 nm. Unused medium samples (blank medium) served as the controls for these assays.

### 2.9. RNA-Sequencing Data Analysis

#### 2.9.1. Preparation of RNA-Seq Libraries

cDNA libraries derived from Poly-A mRNA were generated using Illumina’s TruSeq RNA v2 Sample Prep Kit. Briefly, Poly-A mRNA was isolated from total RNA samples (0.2–1 μg input) using hybridizaton to Poly-T beads. Poly-A mRNA was fragmented at 94 °C in the presence of divalent cations, followed by first-strand cDNA synthesis using random hexamers and SuperScript IV reverse transcriptase (Invitrogen, Waltham, MA, USA). Following second-strand cDNA synthesis, end repair, the addition of a single A nucleotide, unique dual indexed adaptor ligation (IDT for Illumina), AMPure bead purification, and PCR amplification, the resulting cDNA sequencing libraries were measured on the LabChip GX, diluted to 3 nM, and stored at −20 °C.

#### 2.9.2. Sequencing

Samples were pooled and clustered on NovaSeq S4 flow cells (24 samples/pool/lane) using on-board clustering. Paired-end sequencing was performed using the XP workflow on NovaSeq6000 instruments with 2∗125 cycles of incorporation and imaging in addition to 2∗8 cycles for the dual indexes. Base-calling was performed in real-time using RTA v3.4.4. The demultiplexing of BCL files and generation of FASTQ files was performed using bcl2fastq2 v.2.20.

#### 2.9.3. Gene Expression Quantification

The RNA transcript expression was quantified with Kallisto version 0.46.1 [19] using default parameters and the Ensembl Homo_sapiens GRCh38 reference transcriptome and generated FASTQ files as input.

#### 2.9.4. Differential Gene Expression Analysis

We used DESeq2 [20] version 1.26.0 to determine statistically significant differentially expressed genes (DEGs) across experimental conditions (*p* < 0.05 and Benjamini–Hochberg correction α = 0.1 adjusted *p* < 0.1). From downstream analysis we filtered out those DEGs that when compared across conditions (i) were lowly expressed (<2 TPM), (ii) showed relatively small fold-change (FC) differences (abs log_2_ FC < 1), (iii) had highly variable expression across biological replicates (relative standard error of the mean > 1/2), and (iv) were inconsistent across donors (significant change for only one of the three patients). This filter resulted in a set of 1106 response genes that we used as input for downstream analysis. We used the resource DoRothEA to annotate DEGs as transcription factors [21]; the human genome-scale metabolic network reconstruction Recon3D to annotate DEGs as metabolic genes [22].

#### 2.9.5. Gene Set Ontology Enrichment Analysis

We used the gene ontology online tool AmiGO 2 (http://amigo.geneontology.org/amigo, accessed on 24 June 2021) to associate statistically significant pathway enrichments to gene sets of interest [23]. We selected the following options: “Reactome pathways” as the annotation data set (Reactome version 65; released 17 November 2020), “Fisher’s exact test” as the test type and “False Discovery Rate” as the multiple test correction. We downloaded results as JSON files and formatted them as tables using custom Python scripts.

#### 2.9.6. Regulatory Influence Inference Algorithm

First, we computed the expression z-score of the set of 1106 response genes. Separate to upregulated and downregulated gene sets, we applied agglomerative clustering (sklearn.cluster.AgglomerativeClustering function) exhaustively evaluating the number of clusters (*k*) ranging from extreme values as low as 3 and as high as 114. Then, we quantified partition goodness using Silhouette, Calinski–Harabasz and Davies–Bouldin scores. Consistently optimal partitions (*k* = 41 and *k* = 35 for upregulated and downregulated gene sets, respectively) defined expression clusters. Next, we probed obtained clusters for enriched regulatory influences defined in DoRothEA regulons using hypergeometric tests [21]. We corrected multiple testing using Benjamini–Hochberg correction α = 0.1. We then merged enriched gene sets that shared a common regulator. Finally, we evaluated if regulator expression profiles correlated (Pearson correlation coefficient > 0.8) with its identified target genes mean expression. We defined such sets as *regulons*. We assigned regulon activity for a particular condition as the gene expression mean over the target genes. We visualized clustered regulon activities in the form of a heatmap using the seaborn.clustermap function.

#### 2.9.7. TF–TF Regulatory Influence Network Visualization

We established a directed edge between two TFs if a regulator (tail) had as the target gene another TF (head). We used Cytoscape [24] version 3.8.2 to visualize the inferred TF–TF influence network applying the hierarchical layout.

#### 2.9.8. Code Availability

All computational methods used for the analysis of RNA-seq data and beyond are available in the GitHub repository https://github.com/adelomana/osteo (accessed on 1 November 2021).

### 2.10. Intracellular Labelled and Unlabeled Metabolite Extraction

In order to extract intracellular metabolites, collected samples (a cell suspension in 80% methanol solution, previously stored at −80 °C) were thawed and 30 µL of previously mixed isotopically-labeled internal standards were then added. After this, the samples were placed on a floating rack and into a sonicating water bath for 20 s sonication before being put on ice for up to 2 min. This was carried out 3 times. Icing prevented the samples from overheating. Subsequently, 800 µL of ice-cold methanol:dH_2_O (7:3 *v*/*v*) solution was added to each sample and samples were vortexed for approximately 30 s. The samples were then centrifuged at 20,817× *g* for 15 min at 4 °C.

The resulting supernatant was then transferred into properly labeled Eppendorf tubes (2 mL) whilst the precipitate was again reconstituted with 900 µL of previously described methanol solution before being vortexed and centrifuged for the second time. The resulting supernatant was combined with the previous one and precipitates retained at −80 °C for the future BCA protein assay (for data normalization purposes).

In two separate sets of twelve Eppendorf tubes, 200 µL of varying dilutions of previously prepared CC mix were transferred. The CC mix, also known as a mixture of metabolite standards, is a dilution series that can be used for the absolute quantification of metabolite concentration by generating an external calibration curve by least-squares linear regression. This curve is then used to estimate the absolute concentrations of the corresponding metabolites in the measured experiment samples.

All samples (CC mix serial dilutions and intracellular samples) were then transferred to a vacuum concentrator (MinVac) for evaporation before being reconstituted in 300 µL of solution containing dH_2_O and ACN in equal parts.

The reconstituted samples, as well as the two CC mixes, were then filtered through a Pierce protein 96-well precipitation plate that had previously been prepared by wetting the filter (to facilitate correct filtration). The filtration was conducted via centrifugation for 30 min at 4 °C and 2000 rpm.

The filtered precipitation was then transferred to a labeled set of glass mass spectrometry vials with glass inserts in order to be put through the mass spectrometer (UPLC-MS).

### 2.11. BCA Protein Assay

In order to evaluate cell quantity at each time point defined by change point analysis, that could then be used to normalize data, a bicinchoninic acid (BCA) protein assay was performed on the precipitates collected during the intracellular metabolite extractions.

In order to extract and dissolve protein content, a dissolving buffer was created. It constituted buffer A (RIPA buffer):buffer B (200 mM NaOH, 1% SDS) in 1:1 *v*/*v*. Then, 100 µL of the dissolving buffer was added to the Eppendorf tubes containing the protein pellets and the volume was adjusted based on the estimated amount of protein. Afterward, the tubes were vortexed and put through a freeze/thaw cycle to aid pellet breakdown. Next, the pellets were sonicated for 1 h at 60 °C before being vortexed again to try and dissolve as much protein as possible. Of the resulting suspension, 5 µL of each sample were used for the assay.

For the performance of the assay itself (Pierce^TM^ BCA Protein Assay), the accompanying kit protocol from Thermo Scientific^TM^ was followed.

### 2.12. UPLC-MS Set Up and Run Configuration

All metabolite measurements based on mass spectrometry were performed using a gradient elution UPLC (ACQUITY) system (UPLC ACQUITY, Waters Corporation, Milford, MA, USA) coupled with an ionization qTOF mass spectrometer (Synapt G2 HDMS, Waters Corporation, Manchester, U.K.) with an electrospray interface (ESI). Briefly, the gradient chromatographic separation of samples was achieved by HILIC through an Aquity BEH amide column (2.1 mm × 150 mm, 1.7 µm particle size, Waters Corporation) at 45 °C. Two different chromatographic conditions were used in combination with the HILIC column, an acidic mobile phase (phase A), and a basic mobile phase (phase B). In both cases the injection volume was 7.5 µL, the flow rate was 0.4 mL/min and the run time was 14 min. Mobile phase A conditions consisted of ACN with 0.1% of formic acid and mobile phase B conditions consisted of dH_2_O with 1% formic acid. The following gradient patterns (solvent B) were used in both cases: 0 min 1% B, 0.1 min 1% B, 6 min 60% B, 8 min 40% B, 8.5 min 1% B, 14 min 1% B. Both positive and negative ESI modes were acquired. The capillary voltage and the cone voltage were 1.5 kV, the temperatures were 120 °C and 500 °C, respectively, and desolvation gas flow was 800 l/h.

### 2.13. Metabolomics Data Normalization and Processing

#### 2.13.1. Extracellular Metabolomic Data

Data processing and normalization was performed in R [25]. One day was found to be missing more than 50% of data points and removed from further analysis. Other data points below the limit of detection or for other reasons, were replaced with the minimum metabolite value measured. For each metabolite measured, a generalized linear model was fitted using the gamm4 package in R [26]. This modeled the change in metabolite concentration by day of differentiation protocol accounting for donor variation and analysis batch effects as random variables. Other possible models were considered, accounting for the media dwell time and PIPL batch nested in donor and passage number nested in donor as random effects. However, the Akaike Information Criteria of these models (see Supplementary Table S2 online) were higher indicating poorer models, with either less good fit or more over fitting. One exception to this was adding the effect of media well time to the lactate model. However, this was only slightly better, and given the desire for consistency across metabolites and the use of rate of change for other analyses, which incorporates this factor, it was decided not to use this model. Change point detection was performed using the ECP package for R to perform change point detection in multivariate data using the E-Divisive method with a required significance level of 0.05 [27]. This analysis was performed on the modeled values for each day after they had been converted to the hourly rate of change (i.e., secretion or uptake rate) for each day.

The hourly rates of uptake and secretion were calculated by taking the difference of the absolute metabolite concentration levels between adjacent days and dividing by the appropriate number of hours that passed between the media change and sample collection.

#### 2.13.2. Intracellular Unlabeled Metabolomic Data

The unlabeled intracellular metabolomic data was run as an untargeted analysis using the R package XCMS. The first step of the process was conversion of the raw LC-MS data files but in order to be able to work with the data outside of MassLynx and TargetLynx it had to be converted to a MZdata format using MassWolf and a custom-built “water.convert.R” function.

After the raw data conversion had been completed the centWave algorithm was used to automatically detect chromatographic peaks. In this analysis three consecutive ROI with no peaks and an intensity level of at least 100 were considered empty and discarded.

Within the centWave algorithm, once all ROIs were found, a continuous wavelet transformation (CWT) was used to detect chromatographic peaks with variable peak widths.

The obiwarp method was used to align retention times between samples and the peak density method to group corresponding chromatographic peaks.

The R package CAMERA was then used to decrease the complexity of the generated data set by grouping ion features such as adducts and fragments that could potentially stem from the same compound.

The resulting data was then normalized using the NOMIS method (function NormQC in R), then by protein content (BCA protein piercing assay to account for cell number), and finally log-transformed. This data was used for the PCA, PLSDA, one way ANOVA, and Tukey’s post-hoc analysis.

#### 2.13.3. Mummichog Pathway and Network Analysis

To search for enrichment patterns in metabolic networks and identify possible characteristic metabolites that were enriched for each stage we applied the Mummichog online software [28]. The analysis was run as described in the protocol that follows the online software.

#### 2.13.4. Intracellular Labeled Metabolomic Data

The labeled intracellular metabolomic data was run as targeted analysis and integration of targeted compound peaks was conducted using TargetLynx (v.4.1, Waters), an application manager. The raw MS data with was then corrected to account for all naturally abundant ^13^C-glucose isotopes via IsoCor [29]. This provided the percentage of isotopes for each molecule that exceeded the natural abundance along with corrected isotopologue distribution.

## 3. Results

First, we verified osteogenic differentiation with well-known biomarkers. Then, we used key metabolites to define the metabolic stages of osteogenic differentiation. Next, we used untargeted and targeted metabolomic analysis to globally characterize these metabolic shifts. Finally, we reconstructed a gene regulatory network to explore the underlying mechanisms of the observed metabolic shifts.

### 3.1. Verification of Osteogenic Differentiation

The determination of osteogenic differentiation was performed for all donors using Alkaline Phosphatase (ALP) activity and Alizarin Red staining. Final differentiation was verified through measurements of transcript levels of *RUNX2*, secreted phosphoprotein 1 (*SPP1*), and collagen type 1 alpha 2 chain (*COL1A2*) (see Supplementary Figure S1A). ALP activity was higher at all sampling timepoints for all donor samples than in the blank medium, though the increase in the average ALP level was only significant at Day 14 (Bonferroni-adjusted two-tailed unpaired *t*-test, *p* = 0.1194 for Day 7, *p* = 0.0117 for Day 14, and *p* = 0.6072 for Day 28). These results follow the typical pattern of ALP activity during osteogenic differentiation [30,31], i.e., initially increasing then plateauing. Measurement values are presented in Supplementary Table S3 online.

Mineralization, in the form of calcium phosphate accumulation, was verified using Alizarin Red staining, both visually (Supplementary Figures S1B and S2) and by calculating a fold-change with respect to the Day 0 control (Supplementary Table S4 online); however, these changes were not statistically significant despite visually apparent accumulative mineralization.

The comparison of *RUNX2* and *COL1A2* expression are shown in Supplementary Table S5 online. *RUNX2* expression increased while *COL1A2* decreased over time.

In summary, ALP activity, mineralization, and gene expression profiling over the 28-day study period all verified that osteogenic differentiation was initiated by the addition of the osteogenic medium (OM).

### 3.2. Lactate Secretion Changes during Osteogenic Differentiation

**Figure 1:**
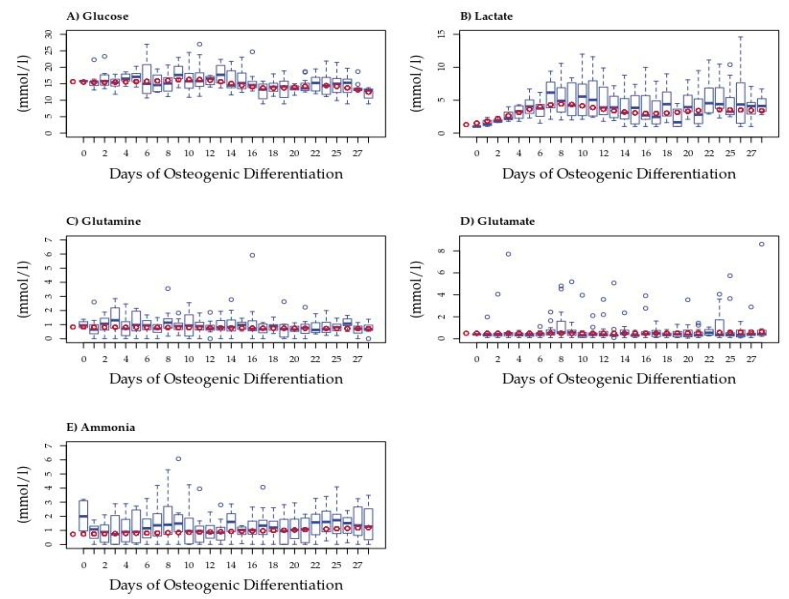
Extracellular metabolite measurements in spent medium from MSCs during osteogenic differentiation. Box plots show concentrations (mmol/l) of (**A**) glucose (*n* = 4), (**B**) lactate (*n* = 4), (**C**) glutamine (*n* = 4), (**D**) ammonia (*n* = 4), and (**E**) glutamate (*n* = 4). Dark blue circles represent outliers. Red circles represent the model predicted value, subsequently used in change point analysis.

To obtain an initial estimate of changes in the central carbon metabolism we quantified extracellular glucose, lactate, glutamate, glutamine, and ammonia concentrations in the spent medium from MSCs undergoing osteogenic differentiation (Figure 1). These rapid detection methods were limited to key metabolites allowing frequent measurements outlining the changes over time.

To account for differences due to inter-donor variation, we fitted a model to these data; model predictions were used in all further analyses. Only extracellular lactate differed significantly from that of the blank medium on Days 19 and 23 (Figure 1B). Average rates of concentration change (mmol/l/hr) each day can be seen in Supplementary Figure S3B. The average amount of lactate measured was significantly higher during the last 13 days of differentiation than in the blank medium, with hourly secretions also higher in those later stages. The concentration changes observed for other metabolites were less apparent. Nevertheless, trends were observed in extracellular glucose that dropped following Day 12 but did not differ significantly from that in the blank medium (Figure 1A). Glutamine was generally consumed in small amounts each day with an increase in consumption over time, possibly due to an increase in cell numbers (see Figure 1C and Supplementary Figure S3C). Glutamate concentration was little changed in the medium at the beginning of the differentiation period, then began to be slightly secreted around Day 15, and showed increasing secretion after Day 21, although the rate of secretion remained low (Supplementary Figure S3D). Ammonia showed an increasing trend toward secretion throughout differentiation, possibly due to variation in cell numbers (Supplementary Figure S3E). Despite variability, generalized linear modeling revealed patterns in the rates of change of these metabolites during osteogenic differentiation. In order to define these patterns, we performed multivariate change point analysis using the normalized data for all five metabolites.

### 3.3. The Utilization and Production of Essential Metabolites Are Indicative of Three Stages of Osteogenic Differentiation

**Figure 2:**
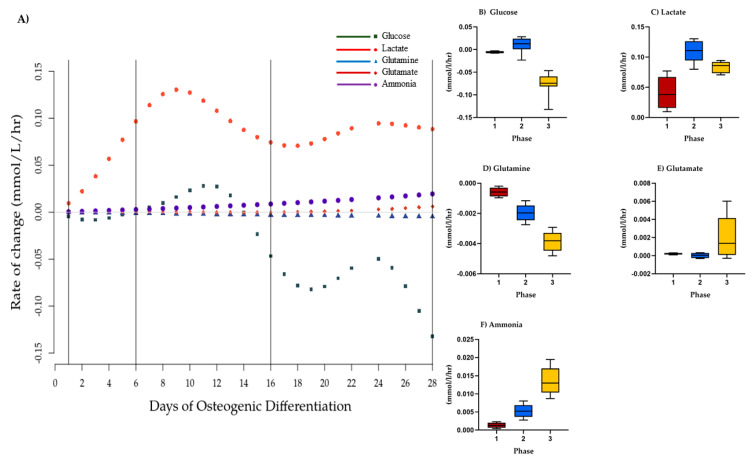
Phases of osteogenic differentiation. (**A**) Change point analysis for glucose, lactate, glutamine, ammonia, and glutamate in spent medium from osteogenically differentiation MSCs between Days 1 and 28. Vertical lines indicate four possible change points separating the stages of differentiation. Points (various shapes) represent the model normalized rate of change on a given day (mmol/L/h). Black lines represent the change points detected at less than *p* = 0.05. Gray line represents 0. Hourly rate of change of concentration of model normalized values (mmol/L/h) per phase of osteogenic differentiation, mean, and SEM of (**B**) glucose, (**C**) lactate (**D**) glutamine, (**E**) ammonia, and (**F**) glutamate. Red = Phase 1, blue = Phase 2, yellow = Phase 3. Differences between phases were not statistically significant.

Multivariate change point analysis was performed using model normalized values of the hourly rate of change for all five metabolites in the spent medium from osteogenically differentiating MSCs from four donors. We identified two statistically significant change points within the differentiation period (*p* < 0.05), in addition to the beginning and end, defining three metabolic stages of differentiation (Figure 2). Phase 1 occurred from Days 1 to 4, Phase 2 was between Days 5 and 15, and Phase 3 was from Day 16 to the end of the study period at Day 28.

We next calculated glucose/lactate ratios within the three phases as a proxy for glycolysis. These ratios were different within the three phases (Table 1). During Phase 1, a glucose/lactate flux ratio of −0.209 was observed that then dropped in Phase 2 before increasing to 0.908 during Phase 3 indicating changes to glycolysis between the three stages. Similarly, glutamine/glutamate and glutamine/ammonia flux ratios were suggestive of changes to glutaminolysis. These analyses suggest different metabolic phases over the 28 days of osteogenic differentiation that are defined by changes to glycolysis and glutaminolysis.

### 3.4. ^13^C-Glucose Mean Label Enrichment Suggests an Anaerobic Switch following Day 9 of Osteogenic Differentiation

**Figure 3:**
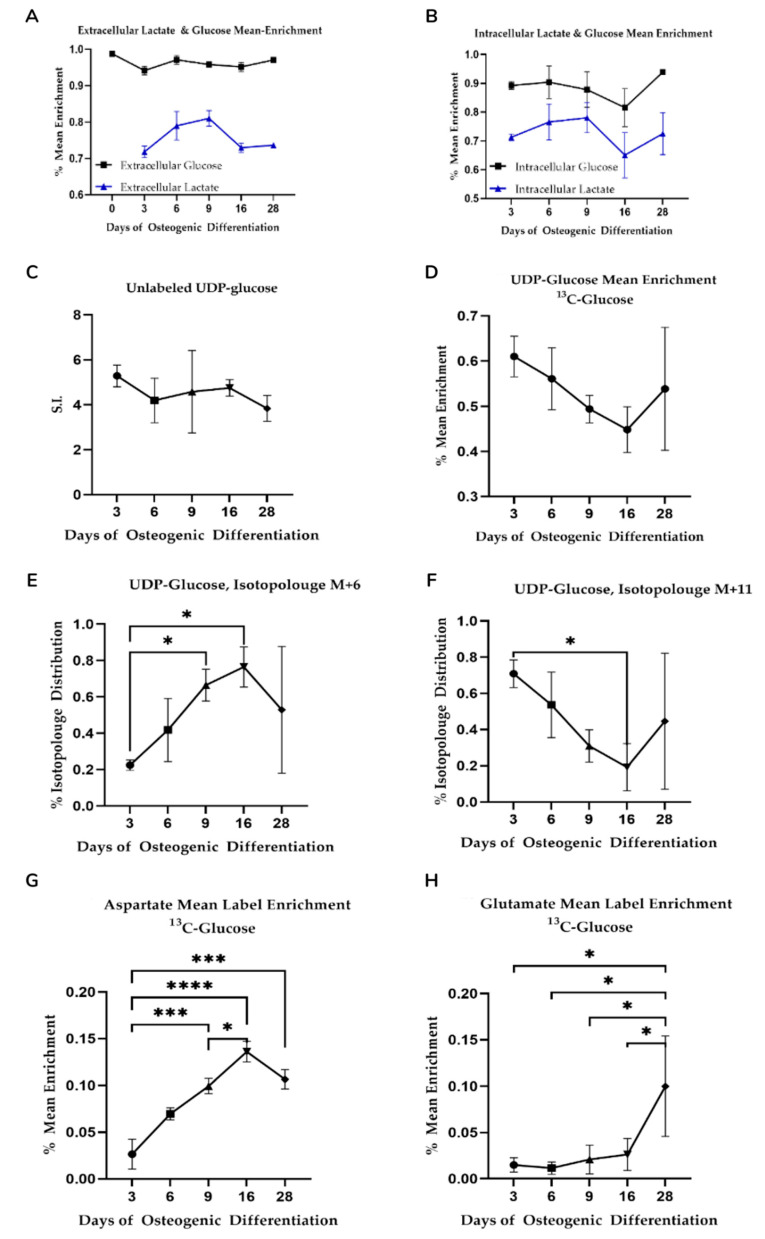
(**A**,**B**) Mean ^13^C-glucose label enrichment in intra-and extracellular glucose and lactate. Shown is average value (*n* = 3) with standard deviation. No statistical significance in mean label enrichment between days was observed. Data is normalized to cell quantity. (**C**–**F**) Isotopologue distribution of UDP-glucose supports increased glycolysis during osteogenic differentiation. Shown is average value (*n* = 3) with standard deviation. Star-marked comparisons are statistically significant at *p* < 0.05. All other cases that are not star-marked are non-significant. S.I. = signal intensity. (**G**,**H**) Mean label enrichment in aspartate and glutamate from ^13^C-labeled glucose. Shown is average value (*n* = 3) with standard deviation. Star-marked comparisons are statistically significant at: * *p* < 0.05; *** *p* < 0.001; **** *p* < 0.0001.

While offering good temporal resolution, our extracellular data lacked sensitivity, therefore, we used uniformly labeled ^13^C glucose-labeling to trace lactate at the defined timepoints to verify that altered metabolic phenotypes during osteogenic differentiation are defined by changes to glycolysis. Label enrichment in glucose was near to 100% (>0.999) and was 95–97% in the spent medium during differentiation (Figure 3A). A trend toward lowered glucose uptake into the cells was observed up until Day 16 although this was not statistically significant. ^13^C lactate, however, increased over the first three time points (going from 71.8% to 81%) and dropped at Day 16 (from 65–72%) indicating an increase and subsequent slow down to glycolysis during differentiation (Figure 3B).

We also observed ^13^C label enrichment from glucose in uridine diphosphate glucose (UDP-glu) that is derived from the glycolytic intermediate glucose-6-phosphate. UDP-glu is substrate for glycogen and glycan synthesis and consists of a pyrophosphate group, pentose sugar ribose, glucose, and the nucleobase uracil. The M + 6 and M + 11 isotopologues of UDP-glu can be used to gain insight into glycolytic and pentose phosphate pathway activity on account of the hexose and pentose moieties, respectively. Specifically, the hexose in UDP-glucose originates from glycolysis and because the only source of glucose available to the cells is from the added ^13^C-labeled glucose in the media, the percentage of isotopologue distribution of the M + 6 isotopologue of UDP-glu is an indicator of glycolytic activity. Similarly, the M + 11 isotopologue of UDP-glu consists of both labeled glucose from glycolysis and the ribose originating from the pentose phosphate pathway (PPP).

UPD-glu concentration fluctuated during differentiation although changes observed were not statistically significant (Figure 3C). Somewhat surprisingly, the mean ^13^C label enrichment (Figure 3D) dropped steadily until Day 16 as opposed to lactate, although these were not statistically significant. Label enrichment into the M + 6 isotopologue, however, increased steadily until Day 16 (Figure 3E) while the M + 11 isotopologue decreased over the same period (Figure 3F) indicative of increased glycolytic flux and decreased PPP, respectively.

Like UDP-glu, UDP-nAcGlu is derived from glycolysis albeit via frucose-6-phosphate and the hexosamine biosynthetic pathway and can be used as a proxy to estimate flux through glycolysis and the PPP. Isotopologue distribution patterns for UDP-nAcGlu mirrored those seen for UDP-glu (Supplementary Figure S4). Combined, the changes observed in lactate excretion during differentiation along with changes to isotopologue enrichment are indicative of glycolytic flux changes and altered flux into peripheral metabolic pathways including glycan synthesis and the pentose phosphate pathway.

### 3.5. Mitochondrial Activity Increases as Differentiation Progresses and Is Significantly Different between Suggested Phases

One reason for changes to glycolytic activity is enhanced reliance on TCA cycle-driven respiration. Mitochondrial activity and aerobic metabolism have been shown to increase as osteogenic differentiation progresses [11,12,15]. To assess the TCA cycle, we investigated ^13^C-glucose-derived label enrichment in aspartate and glutamate, incorporated via oxaloacetate and α-ketoglutarate.

We observed an increase in ^13^C incorporation into aspartate with time prior to a slight drop between Day 16 and Day 28 (Figure 3G) consistent with an overall increase in glucose-derived carbon flux via oxaloacetate. We observed significantly different enrichment changes between the proposed three differentiation phases (*p* < 0.01 for Day 3 vs. Day 9, 16, and 28 comparisons). With respect to glutamate label incorporation, we found an increasing trend, particularly on Day 28 (Figure 3H). Label incorporation was different for the proposed metabolic phases (see Supplementary Table S6 for all comparisons). Combined, aspartate and glutamate enrichment patterns are indicative of changes to TCA cycle flux concomitant with the decrease in glycolysis as osteogenic differentiation progresses.

**Figure 4:**
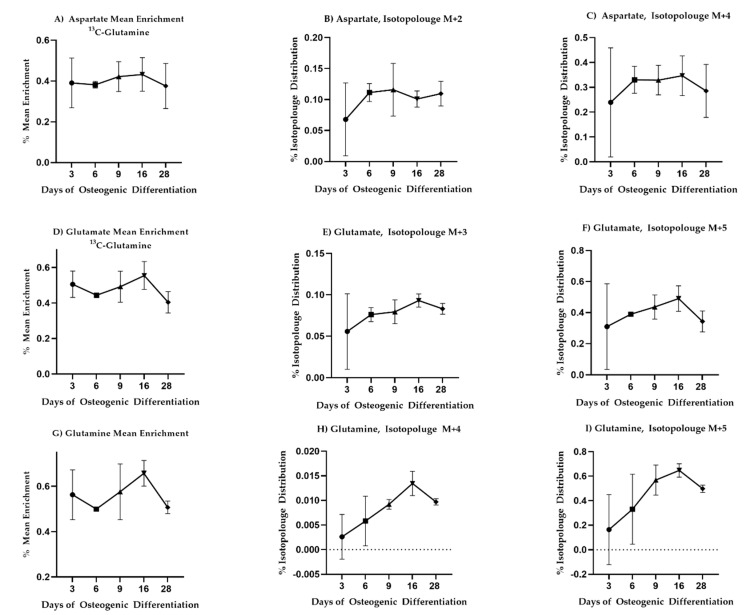
^13^C-glutamine enrichment in glutamate, aspartate, and glutamine is consistent with increased need for extracellular glutamine as differentiation progresses. Mean enrichment and different isotopologue trajectories for different metabolites. All comparisons came across as non-significant. Mean and standard deviation are shown for *n* = 3 samples.

To investigate if these changes were associated with altered glutaminolysis as hypothesized from the changepoint analysis, we fed the cells with uniformly labeled ^13^C or ^15^N glutamine and again traced label incorporation to glutamate and aspartate. The mean enrichment of ^13^C label in intracellular glutamine was quite variable and no statistically significant changes in label enrichment were observed during differentiation (Figure 4). Nevertheless, a trend in label enrichment in the m + 5 isotopologue of glutamine increased toward Day 16 (Figure 4I). This trend was mirrored in the m + 5 isotopologue of glutamate (Figure 4F) and coincided with a drop in the total intracellular concentrations of glutamine and glutamate (see Supplementary Figure S6G,H), consistent with enhanced demand for extracellular glutamine. ^15^N label incorporation into glutamate and aspartate similarly increased on Day 16 consistent with altered glutaminolysis (see Supplementary Figure S5).

### 3.6. Intracellular Metabolomics Validate Metabolic Stages of Osteogenic Differentiation

To confirm results from the change point analysis and screen for additional changes in metabolism, we performed untargeted intracellular metabolomics at five different time points based on previous results (*n* = 3 in all instances).

**Figure 5:**
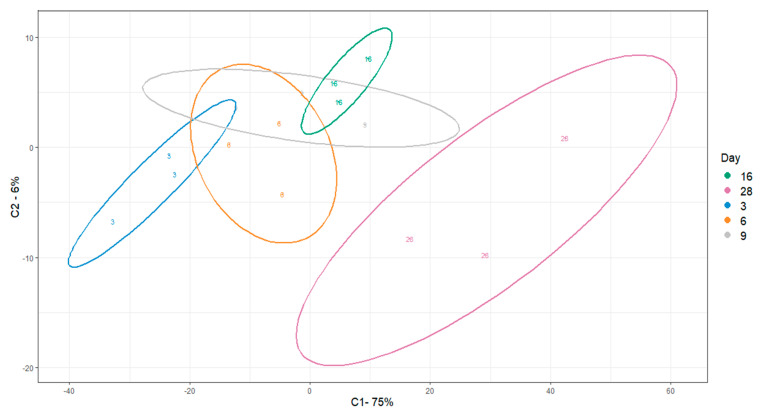
PLSDA visualization of differential *m*/*z* features across osteogenic differentiation. Ellipses indicate 95% confidence intervals.

Untargeted peak identification yielded a total 1682 *m*/*z* features. One-way ANOVA statistical analysis yielded a total of 1064 *m*/*z* features that were significantly different (*p* < 0.05) between at least one of the comparisons of interest. After using Tukey‘s HSD post-hoc test (THSDpht) analysis to correct for multiple comparisons, the total number of significant metabolites (*p* < 0.05) was reduced to 1041 *m*/*z* features (Supplementary Table S3). We visualized sample differences using these features by PLSDA (Figure 5). Separation with time was captured by principal component 1 (PC1). With respect to the three proposed metabolic phases from the change point analysis, grouping was observed between Day 9 (Phase 2) and day 28 (late Phase 3). Day 9 (Phase 2), however, overlapped with Day 16 (start of Phase 3). A clear separation between Day 3 and Day 6 (Phase 1) with respect to Day 28 was also seen. A slight overlap between Day 6 and Day 16 was also observed. In general, the results support different continuous metabolic phases during osteogenic differentiation.

**Figure 6:**
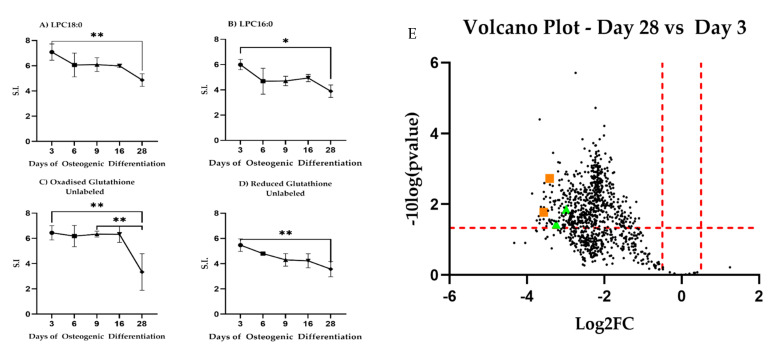
High confidence annotated differential *m*/*z* features. Changes in absolute intracellular concentration levels for (**A**) LPC18:0, (**B**) LPC16:0, and (**C**,**D**) glutathione during differentiation. All metabolites show changed levels across the time observed. * Indicates *p* < 0.05; ** indicates *p* < 0.01. S.I. = Signal Intensity. (**E**) Volcano plot showing log_2_ fold-changes (log2FC) for intracellular untargeted metabolites between Day 28 and Day 3 during osteogenic differentiation. LPC18:0, LPC16:0 shown as green triangles, glutathione as orange squares.

We next associated identified *m*/*z* features with metabolic pathways and specific metabolites using the Mummichog algorithm [28]. Out of 1041 significantly changing *m*/*z* features, we found a core set of 57 *m*/*z* features able to discriminate comparison over the three determined phases. Additionally, we attempted to annotate the 1041 significant *m*/*z* (Supplementary Table S7) features by manually validating the computationally proposed Mummichog annotations by comparison to an in-house spectral library with respect to peak retention time and MS/MS fragmentation. The Mummichog algorithm provided annotations for 22 of these *m*/*z* features (Supplementary Table S8). A total of four *m*/*z* features could be validated with high confidence corresponding to lysophosphatidyl choline (LPC) 16:0, LPC 18:0, and oxidized and reduced glutathione (Figure 6).

**Figure 7:**
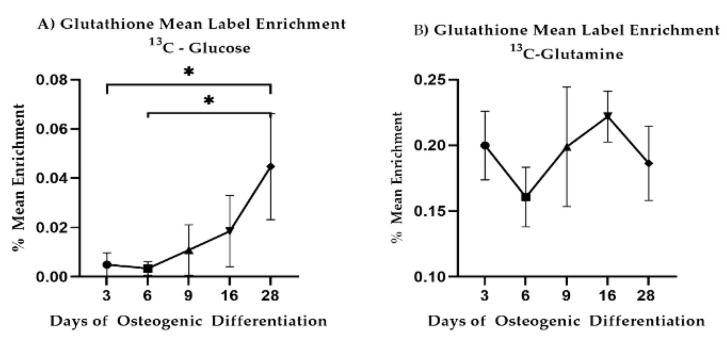
^13^C-glucose and ^13^C-glutamine mean label enrichment in intracellular glutathione. Mean and standard deviations are shown for *n* = 3 samples. Star-marked comparisons are statistically significant at: * *p* < 0.05.

LPC18:0 decreased between Day 3 and Day 28 (*p* = 0.0083) similar to LPC16:0 (*p* = 0.0131). Reduced glutathione decreased steadily between Day 3 and Day 28 (*p* = 0.0052) while oxidized glutathione dropped at Day 28 as compared to Day 3 (*p* = 0.079) and Day 9 (*p* = 0.0099). Given these results, we revisited our ^13^C-labeled glucose and ^13^C-labeled glutamine precursor experiments focusing on glutathione. We observed that glutathione synthesis from glucose increased during differentiation (Figure 7A). Carbon contribution from glutamine remained stable (Figure 7B). Combined, these results are indicative of altered lipid metabolism and changes to redox potential and are consistent with changes to mitochondrial respiration during differentiation.

### 3.7. Gene Expression Analysis Indicates Major Changes in Cellular Function during Osteogenic Differentiation

To better understand the molecular mechanisms behind osteogenesis and the observed metabolic changes, we sought to quantify which transcriptional changes occurred along with this transition.

We sampled cell cultures on Day 3, 6, 16, and 28 for RNA sequencing. We performed transcriptome quantification and gene differential expression calling with kallisto and DESeq2, respectively [20,32]. Furthermore, we filtered out differentially expressed genes (DEGs) that (i) were lowly expressed, (ii) showed relatively small fold-change (FC) differences, (iii) had highly variable expression across biological replicates, and (iv) had inconsistent expression between donors—see Methods for details—resulting in a total set of 1106 DEGs across all experimental conditions (Supplementary Table S9).

**Figure 8:**
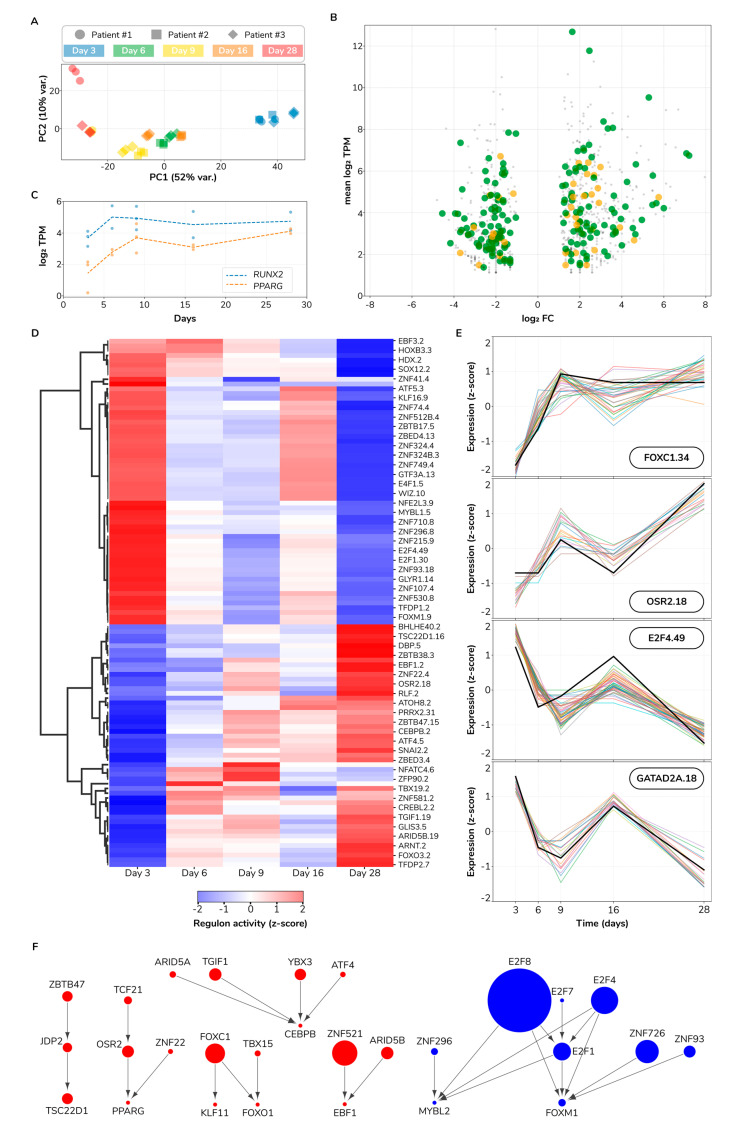
Gene regulatory influence inference reveals a transcriptional switch in osteogenic differentiation. (**A**) Principal component analysis projection of sampled transcriptomes. PC, principal component. (**B**) Scatter plot of differentially expressed genes between last (Day 28) and first sampled (Day 3) time points. Transcription factors are highlighted as large orange dots; metabolic genes as large green dots. FC, fold-change; TPM, transcripts per million. (**C**) Expression profiles for *RUNX2* and *PPARG*. (**D**) Regulon activity heatmap. (**E**) Expression profile of selected regulons. Each line depicts a gene expression profile: black thick lines correspond to the regulon TF, other lines correspond to regulon target genes. (**F**) TF–TF network. Each node represents a TF. Node size maps to the number of target genes of a particular regulator. Red and blue colors indicate transcriptional upregulation and downregulation, respectively. Directed edges denote inferred transcriptional influences.

We sought to investigate the major sources of variation in the set of DEGs. We used principal component analysis (PCA) to project all samples into the two principal components (PCs) that maximized data variance. We found that differentiation time strongly associates with PC1 (52% of total variance), indicating that the differentiation trajectory is the major experimental source of transcriptome variation (Figure 8A). PC2 (10% of total variance) discriminates samples from two donors on the last differentiation time point (Day 28), indicative of a smaller divergence in cell differentiation trajectories between these two donors. Therefore, we conclude that cell differentiation is the major source of transcriptome variation and that trajectory divergence between two particular donors accounts for a smaller secondary source of variation.

Next, we pursued characterizing the transcriptional changes associated with the differentiation process. Toward this goal, we identified the set of DEGs and their associated cellular functions between the last and first sampled time points. In the later stages of differentiation, we found 488 upregulated DEGs of which 25 are transcription factors (TFs) and 81 metabolic genes (MGs). Similarly, we found a total set of 453 downregulated DEGs containing 12 TFs and 81 MGs (Figure 8B and Supplementary Table S10). We further explored which cellular pathways are associated with DEGs using gene set ontology enrichment analysis (see Methods).

The top three most upregulated TFs are *FOS*, *RORB,* and *NR1D1* with a fold-change of 54, 24, and 15, respectively. Other key upregulated TFs are *PPARG*, *BCL6*, *FOXO1*, *CEBPD,* and *NFYB*. Different subsets of these TFs associate with enriched ontologies along three main cellular axes: inhibition of cell death (FOXO-mediated transcription of cell death genes), signaling (interleukin-4 and interleukin-13 signaling), and metabolism (regulation of lipid metabolism by PPARα). Supplementary Table S11 contains all found enriched ontologies. With respect to metabolic genes, the top three most upregulated genes are *ADH1B*, *APOE,* and *MAOA* with a fold-change of 52, 50, and 36, respectively. Other upregulated genes encoding influential enzymes are *PTGDS*, *GPX3*, *PDK4*, *SOD2,* and *DGAT2*. In this gene set, ontology analysis revealed enriched functions revolving around lipid metabolism (chylomicron clearance, steroid hormone, arachidonic acid, and fatty acid metabolisms), compound functionalization, biological oxidation, and iron uptake and transport. In addition to TFs and metabolic genes, we identified an upregulated set of 382 other genes. Examples of this set are *IGF2*, *IL1RL1*, *FGF7*, and *TLR3* with a fold-change of 37, 36, 16, and 2, respectively. We would highlight four enriched functions in this set: degradation of the extracellular matrix, interferon and insulin-like growth factor signaling, and the regulation of the complement cascade. In summary, this set of 488 upregulated DEGs reflects the profound biological shift occurring during the process of osteogenic differentiation.

Just as functionally important as gene upregulation is gene downregulation. Thus, we also determined which genes and biological functions are repressed between the late and early stages of differentiation. The top three downregulated TFs are *MYBL2*, *FOXM1,* and *E2F1* with a fold-change of 13, 9, and 8, respectively (Supplementary Table S10). Other key downregulated TFs are *OSR1*, *HMGA2,* and *DNMT1*. The last two genes encode proteins that regulate chromatin accessibility which suggests ongoing chromatin rearrangements. Furthermore, *OSR1* is a direct target of RUNX2 [33] and its negative regulation is key for osteoblastic differentiation [34]. Regarding metabolic genes, the top three most downregulated genes are *RRM2*, *PKMYT1,* and *UBE2C*, with a fold-change of 21, 17, and 16, respectively. Collectively, cell cycle-related functions are overwhelmingly enriched in this set of downregulated genes: G2/M transition, mitotic anaphase, regulation of mitotic cell cycle, DNA replication initiation, and centrosome maturation are just a few examples of the biological functions significantly enriched in the set of repressed genes in the later stages of differentiation (Supplementary Table S11). Selected examples of downregulated genes that align with these functions are *CDK1*, *CDC25A,* and *POLE*. Furthermore, we noted other enriched functions, e.g., metabolism of carbohydrates, reduction of cytosolic Ca^2+^ levels, and solute-carrier mediated transmembrane transport. Particularly interesting is the downregulation of *SLC25A10*, a gene that encodes the mitochondrial dicarboxylate carrier, which transports dicarboxylates such as malonate, malate, and succinate across the inner mitochondrial membrane, thus, potentially regulating key metabolic pathways like gluconeogenesis and fatty acid biosynthesis, among others. Aside from TFs and metabolic genes, we identified a downregulated set of 360 other genes. This set is also consistently enriched in functions related to cell cycle progression. Moreover, other key factors of this set known to be implicated in osteogenic differentiation are *IL6* and *CXCL12* [35,36]. In summary, this set of 453 downregulated DEGs reflects a substantial reduction in cell cycle progression during osteogenic differentiation.

Fate decision of mesenchymal stem cells to either adipocytes or osteoblasts is controlled by many factors including the interplay between RUNX2 and PPARγ [37]. Consequently, we sought to quantify the temporal expression profile of *RUNX2* and *PPARG*. We visualized both *RUNX2* and *PPARG* expression levels across time (Figure 8C). We found that the *PPARG* expression pattern as increasing, starting from 4 transcripts per million (TPM) on Day 3, to 17 TPM on Day 28 (adjusted *p* < 2 × 10^−12^ and adjusted *p* < 5 × 10^−12^ for two donors, respectively). *RUNX2* instead, while it also increases from 14 TPM to 27 TPM, its temporal profile is mainly flat with a sharp increase from Day 3 to Day 6. *RUNX2* Day 28 vs. Day 3 contrast is significant only for one donor (adjusted *p* = 6.00 × 10^−2^ and adjusted *p* = 6.44 × 10^−3^ for two donors, respectively) and did not pass our fold-change filter (abs log_2_ FC > 1). Altogether, while both genes have a higher expression in the late stages of our experiment, the increasing trend for *PPARG* is more considerable than for *RUNX2*. This observation is in contradiction with the role of RUNX2 as a determinant of osteogenic fate in mesenchymal stromal cells and PPARγ as a counterpoint determining adipogenic fate [38,39]. We propose several possible scenarios to reconcile this observation: (1) *RUNX2*/*PPARG* transcriptional toggle switch occurred much earlier than Day 3 and, therefore, we observed a stage already committed for these two master regulators, (2) RUNX2/PPARγ activity may be predominantly controlled by post-transcriptional modifications, e.g., MAPK-dependent phosphorylation status [40], and (3) changes in other activating/repressing factors. For example, we found the upregulation of both *CEBPB* and *CEBPD*, which are vital for both adipogenic [41,42] and osteogenic identity [43,44]. In parallel, two PPARγ coactivators are differentially expressed, but in opposite directions: *PPARGC1A* is upregulated (from 0 to 8 TPM; log_2_ FC = 3.17) and *PPRC1* is downregulated (from 30 to 7 TPM; log_2_ FC = −1.95). These findings suggest that mesenchymal stromal cells commitment to either osteogenic or adipogenic fates is a rather complex decision involving multiple molecular regulators and interactions at various regulatory levels, ranging from genomic to transcriptional and post-translational mechanisms.

### 3.8. Regulatory Influence Inference Reveals a Cell State Switch

Gene regulatory influence inference methods identify regulators of transcriptional response [45]. We took such an approach to infer which regulators, in this case, transcription factors (TFs), control the observed transcriptional changes during osteogenic differentiation. First, we determined regulon memberships. We defined a *regulon* as a set of expression coherent target genes that all share a binding site for a given TF and collectively correlate to such TF expression (see Methods). We identified 111 regulons involving 111 TFs and 469 corresponding target genes. We observed a typical regulon size of 9 target genes, ranging from as low as 2 and up to 123 target genes. We assigned regulons identifiers using the name of the regulator plus the number of target genes separated by a dot, e.g., our largest regulon, E2F8.123, consists of 123 target genes regulated by the transcription factor E2F8. All regulon memberships including their activity across sampled differentiation time points are provided as Supplementary Table S12. We visualized regulon activity over time (Figure 8D). We observed an evident switch pattern where a set of 60 regulons shifted from active to inactive while 51 other regulons followed the inverse transition. More than 50% of upregulated regulon target genes are controlled by just six TFs: ZNF521, FOXC1, PRRX2, TGIF1, ARID5B, and OSR2. ZNF521, the controller of the largest upregulated regulon ZNF521.45, is a negative regulator of the adipogenic differentiation fate in mesenchymal stem cells [46]. FOXC1, PRRX2, TGIF1, and OSR2 are all known regulators of osteogenic differentiation [47,48,49,50]. However, ARID5B had not been yet implicated as a regulator of osteogenic differentiation to the best of our knowledge. Regarding the downregulation response, just three E2Fs (E2F1, E2F4, and E2F8) control up to 59% of all downregulated regulon target genes. E2Fs are well-known regulators of cell cycle progression, apoptosis and DNA synthesis [51], which aligns well with our previous findings from the ontology analysis on downregulated DEGs (Supplementary Table S11). E2Fs role in specifying either osteocyte or adipocyte cell fates is still unclear with positive and negative influences from different family members at different stages of differentiation which complicates a simple interpretation of this finding [52,53]. Furthermore, we show in Figure 8E selected examples of regulon profiles across time including the TF and their target genes expression. The activity profiles of only three regulons do not strictly match a switch pattern: NFATC4.6, POU6F1.3, and ZFP90.2 display an early strong upregulation with a slower relaxation to near basal levels. From a systems perspective, we observed a fairly ordered system at both early and late differentiation time points, with regulon activity constrained to either on or off states. On the contrary, intermediate time points manifest a much higher system entropy with regulons exploring a broad range of activity levels. This pattern is consistent with a critical state transition before a bifurcation point [54] and has been experimentally observed in other differentiation systems [55]. Overall, regulatory influence inference revealed a transcriptional state switch in osteogenic differentiation of mesenchymal stromal cells.

TFs regulate target genes which may also be TFs, potentially establishing a network of regulatory influences with simple but functionally rich network structures like network motifs [56]. In order to determine the network structure of regulatory influences in the osteogenic differentiation process, we considered a directed edge between two given TFs *A* and *B* if *A* (as the node tail) is the TF of a regulon and *B* (as the head node) is one of its target genes. We built a network of 28 TFs and 26 TF–TF regulatory influence interactions which collectively control 303 target genes (Figure 8F). We observed regulatory cascades, e.g., ZBTB47 → JDP2 → TSC22D1; nodes integrating information from multiple upstream nodes, e.g., CEBPB integrates regulatory influences from four other TFs; subnetworks very close to bi-fans, as FOXC1, TBX15, KLF11, and FOXO1 are one interaction away from the bi-fan motif; and denser and more complex interacting subgraphs, e.g., the downregulation response influence component with nodes integrating information from multiple nodes and concurrently regulating various other nodes. Conclusively, we found a rich network of regulatory influences controlling the osteogenic differentiation of mesenchymal stromal cells. This network has the potential to inform future perturbation experiments to validate the identity of presented master regulators, ultimately facilitating the control of the differentiation process.

## 4. Discussion

### 4.1. Increase in Mitochondrial Activity following Day 9

Through analysis of a limited number of extracellular metabolites, change point analysis suggested two metabolic switches to be in place over the course of 28 days of osteogenic differentiation. Subsequent untargeted analysis of intracellular metabolites supported this initial hypothesis. We observed a clear distinction between Day 3, 9, and 28. However, there was some degree of overlap between Day 3, 6, and 9 (Figure 5), suggesting there are similarities in the early phases, in accordance with other reports of metabolic behavior of MSCs during osteogenic differentiation [57]. The clustering of Day 16, with the first three time points, is unexpected and warrants future investigation with higher temporal resolution sampling around Day16.

Elaborating on these results with more detail, labeling experiments enabled us to identify one clear switch following Day 9. The labeling results confirmed changes in glycolysis and increased mitochondrial activity, with cells diverting more glucose into the TCA cycle, indicative of a metabolic switch. This shift into aerobic metabolism has been shown in several other studies [11,58]; however, the shorter time window identified in this paper can help focus future studies to identify ways to enhance osteogenesis of MSCs and specific timing to apply such treatments.

For example, it has been shown that mitochondrial dysfunction impairs osteogenesis, increases the activity of osteoclasts, and so can accelerate age-related bone loss (i.e., osteoporosis) [59]. A similar observation has been made regarding osteoarthritic (OA) human articular chondrocytes (a related cell type). There, mitochondrial mass within OA chondrocytes increased whilst a higher proportion of the cells had de-energized mitochondria, with a reduction in activity of complexes II and III observed when compared to normal chondrocytes [60]. Further, coenzyme Q10 has been observed in research to inhibit aging of MSCs [61,62] and to improve mitochondrial function and so relieve disease symptoms [63,64]. Taken together with our results it might, thus, be a particularly relevant follow up to research the supplementation of Coenzyme Q10 around day 9 during osteogenic differentiation in order to see if the differentiation of MSCs could be enhanced via increased mitochondrial function.

### 4.2. Increased Glycolysis followed by Decreased PPP Flux as Differentiation Progresses

We observed a lowered flux through the PPP in MSCs during osteogenic differentiation. This was indicated by labeling experiments, specifically the temporal changes in isotopologue distribution differences between M + 6 and M + 11 isotopologues for ^13^C-glucose-labeling for UDP-glucose (Figure 3E,F) and N-acetyl-UDP glucosamine (Supplementary Figure S4); both indicated a similar behavior. In both instances the label enrichment into the M + 6 isotopologue (derived from glycolysis) increased temporally whilst label enrichment into M + 11 (which consists of glucose from glycolysis and ribose derived from the PPP) decreased in a significant manner during that same time period, indicating decreased label enrichment coming from the M + 5 isotopologue and, therefore, a decreased flux through the PPP.

It has been shown that for proliferating cells, the activation of glycolysis accompanied by high activity of PPP is the preferred metabolic state [65], and as the PPP is responsible for generating NADPH that is used in reductive biosynthesis (e.g., fatty acid and ribose synthesis), its active flux can be reasoned to be necessary for MSC proliferation. Furthermore, it is known that MSCs undergoing osteogenesis form multiple cell layers [57], reporting a rapid cell proliferation occurring over the course of the first week before stopping by the end of Week 2. This would indicate a necessity for the maintenance of high flux through the PPP at least for the first seven days of differentiation which goes directly against what our label incorporation results indicate. There, flux through glycolysis increases (shown by glucose label incorporation) whilst, seemingly, flux through the PPP decreases. Despite this decrease, the total protein amount measured in our BCA assays increased during the differentiation period. Based on these findings and the way they seem to contradict results reported in the already mentioned papers, it suggests that further research into the temporal changes to the PPP and its role in osteogenic differentiation and MSC proliferation is necessary.

### 4.3. Temporal Changes in Glutaminolysis and TCA-Cycle Activity

We determined a shift in the redox balance during differentiation with both oxidized and reduced glutathione decreasing over time.

Glutathione plays an important role when it comes to the ability of cells to manage oxidative stress, it is important for the proper function of oxidative phosphorylation (and, thus, mitochondrial respiration) and it is reliant upon glutaminolysis. Glutamine, and subsequently, glutaminolysis has been shown to be essential for successful continued osteoblastogenesis [66]. Glutathione levels are additionally linked to the activity level of the PPP, but the PPP provides NADPH that enables the maintenance of the reduced form of glutathione and so aids in managing oxidative stress. It, therefore, follows that reduced PPP activity is accompanied by the observed reduction of intracellular glutathione, specifically the reduced form (GSH).

When reanalyzing ^13^C-glucose label incorporation with respect to glutathione, label incorporation increased as differentiation continued to indicate an increased synthesis of glutathione from glucose derived carbons—supportive of the earlier results of an increased glycolytic flux compared to flux through the PPP. In conjunction with results for both ^13^C- and ^15^N-labeled glutamine label incorporation into glutamate and aspartate, we inferred an altered glytaminolysis and an increased flux into the TCA cycle as had been suggested [11,67]. This all suggests the increased involvement of mitochondrial respiration which has also been reported as essential for successful osteogenesis.

### 4.4. Unexpected Changes in Lipolysis Suggest Further Investigation

The metabolic pathway analysis revealed a surprising result, that changes to lipolysis during differentiation were observed from the untargeted metabolomics analysis (Figure 6). While further additional lipidomic quantification is outside the scope of this work, our results suggest the need for further investigation of lipid metabolism during osteogenic differentiation. Indeed, it has been reported that normal skeletal homeostasis requires lipids for energy and so mitochondrial long-chain fatty acid oxidation must be active [68].

### 4.5. Upregulated Lipid Metabolism and Downregulated Cell Cycle-Related Anabolic Activity Observed via DEGs

Gene expression analysis lent further support to the notion of distinct cell metabotypes existing throughout the differentiation period. The clearest source for the transcriptome variation observed was the differentiation stage, but many metabolic genes followed this transcription pattern. In particular, the upregulation observed in the regulation of lipid metabolism on top of the changes in lipolysis gained from metabolomic data highlights the apparent importance of fatty acids as a source of energy for successful osteogenesis [68].

The apparent mismatch between measured protein content (cell mass) throughout the differentiation and the observed downregulation of cell cycle-related activity and the matching decrease in flux through pathways connected to proliferation also requires further validation. Linking the DEGs with observed changes in metabolite concentrations and metabolic flux to see how coherently gene expression matches enzyme and subsequent protein/metabolite activity may help to identify perturbation experiments that may result in an actual desired phenotypic change.

Finally, the regulatory network inferred in this paper both highlights the complexity involved in the control of osteogenic differentiation and the importance of the accurate identification of master regulators.

## 5. Conclusions

In conclusion, the changes observed in our study support distinct differences between early- and late-stage osteogenesis on both the metabolic and transcriptomic level: there is an initial preparatory and proliferation stage, coinciding with high ALP activity, while later there is a plateau stage with lower ALP activity. Our metabolomic analysis further supports these two stages, and preliminary results using differential gene expression analysis indicate the possibility of three stages with a transcriptional state flip occurring between the initial and last steps. Future functional work will be needed to fully define the relationships between transcriptomic, metabolic, and functional changes. The current experiments also showed a high degree of donor variability, with respect to both metabolism and osteogenic outcome. Further work to understand these differences will hopefully lead to the identification of metabolic biomarkers or master regulators for successful osteogenesis, and possibly to interventions for increasing the success rate at the donor selection stage. Furthermore, it may be interesting to explore the observed lipolytic changes and related mitochondrial activity in a continuous manner around suggested time points of interest through such technology as Seahorse [69,70] assay and to explore if by impacting the mitochondria in a positive manner can lead to faster progression of osteogenesis. Such experiments would improve our understanding of how metabolism impacts the ability of cells to differentiate.

## Figures and Tables

**Table 1:** Key metabolite secretion/uptake ratios for all 4 stages of osteogenic differentiation as identified through change point analysis. Mean values are shown for all days in each phase normalized values. Expected perfect ratios (EPRs) are: glucose/lactate EPR = −0.5 with secretion of lactate and uptake of glucose; glutamine/glutamate EPR = −1 with higher number associated with higher use of glutamate, and glutamine/ammonia EPR = −1, with secretion of ammonia and uptake of glutamine. A negative sign is indicative of (1) lactate secretion and glucose uptake from the medium, (2) glutamate uptake at a high rate and secretion of glutamine.

Phase	Glucose/Lactate	Glutamine/Glutamate	Glutamine/Ammonia
1 (Days 1–4)	−0.209	−2.620	−0.444
2 (Days 5–15)	0.088 *	−38.299	−0.374
3 (Days 16–28)	−0.908	−1.843	−0.284

* Glucose and lactate were measured as secreted over this phase.

## Data Availability

All data are available upon request.

## Supplementary Materials

The following are available online at https://www.mdpi.com/article/10.3390/bioengineering8120208/s1, Figure S1: Indicators of osteogenic differentiation. (A) Alkaline phosphatase (ALP) activity for Days 0, 7, 14 and, 28. ALP is shown as nmol/min of p-nitrophenyl phosphate converted into p-nitrophenol. (B) Alizarin Red quantification for Days 0, 14, and 28. Mean and standard error of the mean (SEM) are shown (*n* = 4), Figure S2: Visual mineralization results obtained using Alizarin Red staining for 3 of the 5 donors on Days 14 and 28 of osteogenic differentiation (stained cells are shown on the left side of each panel, *n* = 3). (A) Donor 1—Day 14, (B) Donor 2—Day 14, (C) Donor 3—Day 14, (D) Donor 1—Day 28, (E) Donor 2—Day 28, (F) Donor 3—Day 28. Related to Figure 1B, Figure S3: Rate of concentration change (mmol/l/hr) in spent medium for extracellular (A) glucose (*n* = 4), (B) lactate (*n* = 4), (C) glutamine (*n* = 4), (D) ammonia (*n* = 4), and (E) glutamate (*n* = 4) for Days 1 to 28 of osteogenic differentiation. Dots (various shapes) represent the model predicted value subsequently used in change point analysis, Figure S4: Isotopologue enrichment of N-Acetyl-UDP-Glucosamine is consistant with incresed glycolysis but decreased PPP activity. (A) Absolute Concentration of N-Acetyl-UDP-Glucosamine in unlabelled intracellular data (*n* = 3, SD), (B) Mean ^13^C-glucose label enrichment (*n* = 3, SD) (C) Average % isotopolouge distribution of the M + 6 isotopolouge for N-Acetyl-UDP-Glucosamine (*n* = 3, SD). Significant difference indicated with *or** (D) Average % isotopolouge distribution of the M + 11 isotopolouge for N-Acetyl-UDP-Glucosamine (*n* = 3, SS). * *p* < 0.05 or ***p* <0.0099. S.I = Signal intensity, Figure S5: ^15^N- glutamine label incorporation and isotopologue enrichment of aspartate and glutamate. (A) Extracellular mean label enrichment of aspartate (*n* = 3, SD), (B) Intracellular mean label enrichment of aspartate (*n* = 3, SD) (C) Average % isotopolouge distribution of the M + 1 isotopolouge for aspartate (*n* = 3, SD) (D) Extracellular mean label enrichment of glutamate (*n* = 3, SD), (E) Intracellular mean label enrichment of glutamate (*n* = 3, SD) (*n* = 3, SD) F) Average % isotopolouge distribution of the M + 1 isotopolouge for glutamate (*n* = 3, SD), Figure S6: Intracellular and extracellular absolute concentration levels for (A) and (E) glucose, (B) and (F) lactate, (C) and (G) glutamine, and (D) and (H) glutamate (*n* = 3, SD in all instances). S.I = signal intensity * *p* < 0.05 or ***p* <0.0099, Table S1: Primer pairs used in RT-qPCR. Forward and reverse primers used for RT-qPCR analysis of SPP1, RUNX2 and COL1A2 with TBP as a referencing gene, Table S2: Akaike Information Criteria from models fitted with the gamm4 function in R, all models are for each metabolite varying by day with the combinations of random effects considered below, Table S3: ALP activities for 4 different donors during 28 days of osteogenic differentiation. SEM is the standard error of the mean. Related to Figure S1A, Table S4: Alizarin Red S staining measurement results (both optical density and percentage increase in relation to Day 0), validating mineralization, for 4 different donors during 28 days of osteogenic differentiation. SEM is the standard error of the mean. Related to Figure S1B, Table S5: Real-time qPCR results showing gene expression levels of RUNX2 and COL1A2. For each transcription factor, a relative expression level (compared to the housekeeping gene, TBP) is shown for Day 0 (the beginning of osteogenic differentiation) and Day 28 (the end of the differentiation). The calculated fold change between Days 0 and 28 is also shown. SEM is the standard error of the mean.
