# Supplementary material for: Metabolic and Transcriptional Changes across Osteogenic Differentiation of Mesenchymal Stromal Cells
Source: Bioengineering (Basel). 2021 Dec 10;8(12):208. doi: 10.3390/bioengineering8120208 (PMC8698318; doi:10.3390/bioengineering8120208)
Supplement: Supplementary file 1 [file bioengineering-08-00208-s001.zip › USINGM~1.pdf]

Supplemental information.

TITLE: Using metabolomics and gene regulatory networks to define metabolic stages during osteogenic differentiation of mesenchymal stromal cells

AUTHOR LIST: Thora Bjorg Sigmarsdottir[1], Sarah McGarrity[1,2], Snaevar Sigurdsson[5], Adrian López García de Lomana[2], James Yurkovich[3], Ottar Rolfsson[2], Olafur Eysteinn Sigurjonsson[*,1,4]

AUTHOR AFFILIATIONS:
[1]School of Science and Engineering, Reykjavik University, Reykjavik, Iceland
[2]Center for Systems Biology, University of Iceland, Reykjavik, Iceland
[3]Department of Bioengineering, University of California San Diego, La Jolla, CA, USA
[4]The Blood Bank, Landspítali – the National University Hospital of Iceland, Reykjavik, Iceland
[5]Biomedical Center, University of Iceland, Reykjavik, Iceland

CORRESPONDING AUTHOR CONTACT INFORMATION:
Dr. Olafur E. Sigurjonsson, M.Sc., Ph.D., Principal Investigator
School of Science and Engineering, Reykjavik University
Menntavegur 1
102 Reykjavik, Iceland
email: oes@ru.is
Tel: +354-543-5523; Mobile: +354-694-9427; Fax: +354-543-5532

SUPPLEMENTAL TABLES

Table S1. Primer pairs used in RT-qPCR. Forward and reverse primers used for RT-qPCR analysis of SPP1, RUNX2 and COL1A2 with TBP as a referencing gene.

Gene	Forward Primer	Reverse Primer
**RUNX2**	5'-CTTCACAAATCCTCCTCCCCAAGT	5'-AGGCGGTCAGAGAACAAAC
**SPP1**	5'-CCCCACAGTAGACACATATGATG	5'-TTCCAACTCCTCGCTTTCCAT
**COL1A2**	5''-CCTTCAATCCATCCAGACCAT	5'-GTGAGAGAGGAGTTGTTGGAC
**TBP**	5'-CAGTGAATCTTGGTTGTAAACTTGA	5'-TCGTGGCTCTCTTATCCTCAT

Table S2. Akaike Information Criteria from models fitted with the gamm4 function in R, all models are for each metabolite varying by day with the combinations of random effects considered below.

	Donor + Analysis Batch	PIPL/Donor + Analysis Batch	Passage/Donor + Analysis Batch	PIPL/Passage/Donor + Analysis Batch	Donor + Media + Analysis Batch	Donor
**Glucose**	1543.795	1545.795	1545.795	1547.795	1545.795	1558.992
**Lactate**	1168.993	1170.993	1170.993	1172.993	1167.776	1227.897
**Glutamine**	587.1644	589.1644	588.3329	591.1644	595.5849	591.5849
**Glutamate**	292.9485	294.9485	293.2039	296.9485	381.4727	377.9853
**Ammonia**	757.6984	759.3254	758.5992	761.3254	820.5771	816.5771

Table S3*.* ALP activities for 4 different donors during 28 days of osteogenic differentiation. SEM is the standard error of the mean. Related to Figure S1A.

Donor	ALP Activity (nmol/min)			
	**Day 0**	**Day 7**	**Day 14**	**Day 28**
D12	0.98	2.08	3.61	1.44
D13	2.80	4.40	4.89	3.35
D14	0.70	3.21	3.77	1.95
D15	1.92	4.49	4.93	5.20
**Mean (SEM)**	**1.60 (0.48)**	**3.55 (0.57)**	**4.30 (0.35)**	**2.98 (0.84)**

Table S4. Alizarin Red S staining measurement results (both optical density and percentage increase in relation to Day 0), validating mineralization, for 4 different donors during 28 days of osteogenic differentiation. SEM is the standard error of the mean. Related to Figure S1B.

Donor	Optical Density after Alizarin Red Staining (-)			% increase in mineralization	
	**Day 0**	**Day 14**	**Day 28**	**Day 14**	**Day 28**
D12	0.099	0.141	0.148	42.4	49.40
D13	0.103	0.155	1.720	50.4	1570
D14	0.127	0.685	4.000	439	3049
D15	1.956	0.908	5.203	-46.4	166
**Mean (SEM)**	**0.571 (0.462)**	**0.472 (0.193)**	**2.768 (1.133)**	**121.35 (108.13)**	**1208.6 (704.06)**

Table S5. Real-time qPCR results showing gene expression levels of RUNX2 and COL1A2. For each transcription factor, a relative expression level (compared to the housekeeping gene, TBP) is shown for Day 0 (the beginning of osteogenic differentiation) and Day 28 (the end of the differentiation). The calculated fold change between Days 0 and 28 is also shown. SEM is the standard error of the mean.

**Donor**	**RUNX2**			**COL1A2**		
	**Relative Expression**		**Fold change**	**Relative Expression**		**Fold change**
	**Day 0[b]**	**Day 28**		**Day 0[b]**	**Day 28**	
D13	2.43	2.68	1.10	0.03	17.35	0.08
D14	0.88	1.95	2.22	207.70	28.13	0.07
D15	1.28	3.69	2.89	379.06	152.20	0.64
**Mean (SEM)**	**1.53 (0.47)**	**2.77 (0.50)**	**2.07 (0.52)**	**195.60 (109.59)**	**65.89 (43.27)**	**0.26 (0.19)**

[b] Measurements of the transcription factors used to determine relative expression levels for day 0 were obtained from a parallel study done over the same time period, in the same environment, and using the same material and cells from the same donors.

Supplemental Figures

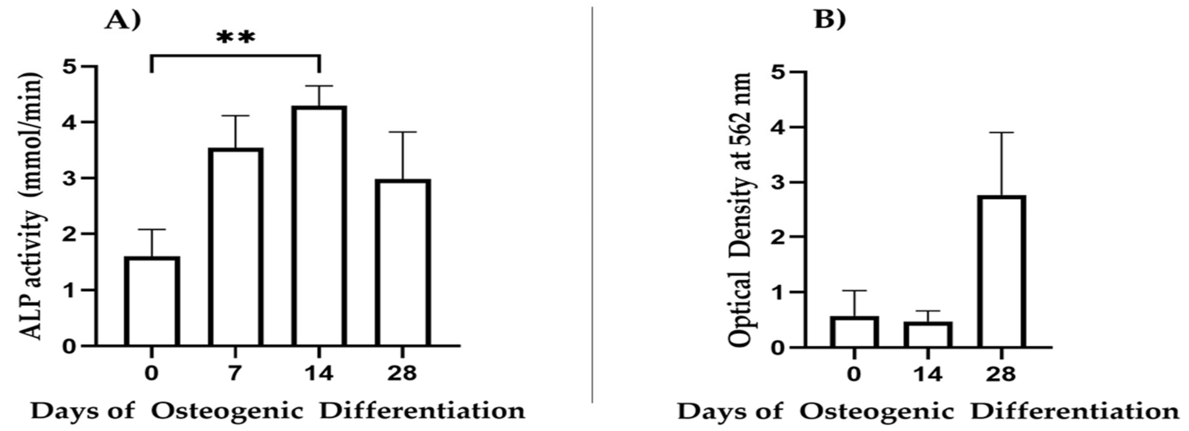

Figure S1. Indicators of osteogenic differentiation*. (*A) *Alkaline phosphatase (ALP) activity for Days 0, 7, 14 and, 28. ALP is shown as nmol/min of p-nitrophenyl phosphate converted into p-nitrophenol.* ***(B)*** *Alizarin Red quantification for Days 0, 14, and 28. Mean and standard error of the mean (SEM) are shown (n = 4).*

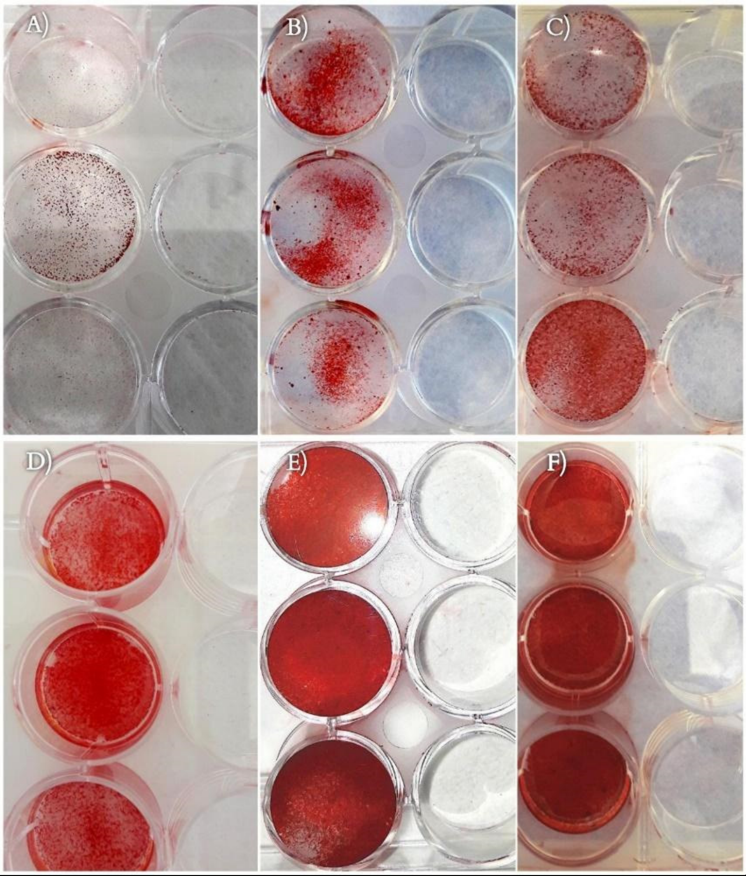

Figure S2. Visual mineralization results obtained using Alizarin Red staining for 3 of the 5 donors on Days 14 and 28 of osteogenic differentiation (stained cells are shown on the left side of each panel, n = 3). A) Donor 1 – Day 14, B) Donor 2 – Day 14, C) Donor 3 – Day 14, D) Donor 1 – Day 28, E) Donor 2 – Day 28, F) Donor 3 – Day 28. Related to Figure 1B.

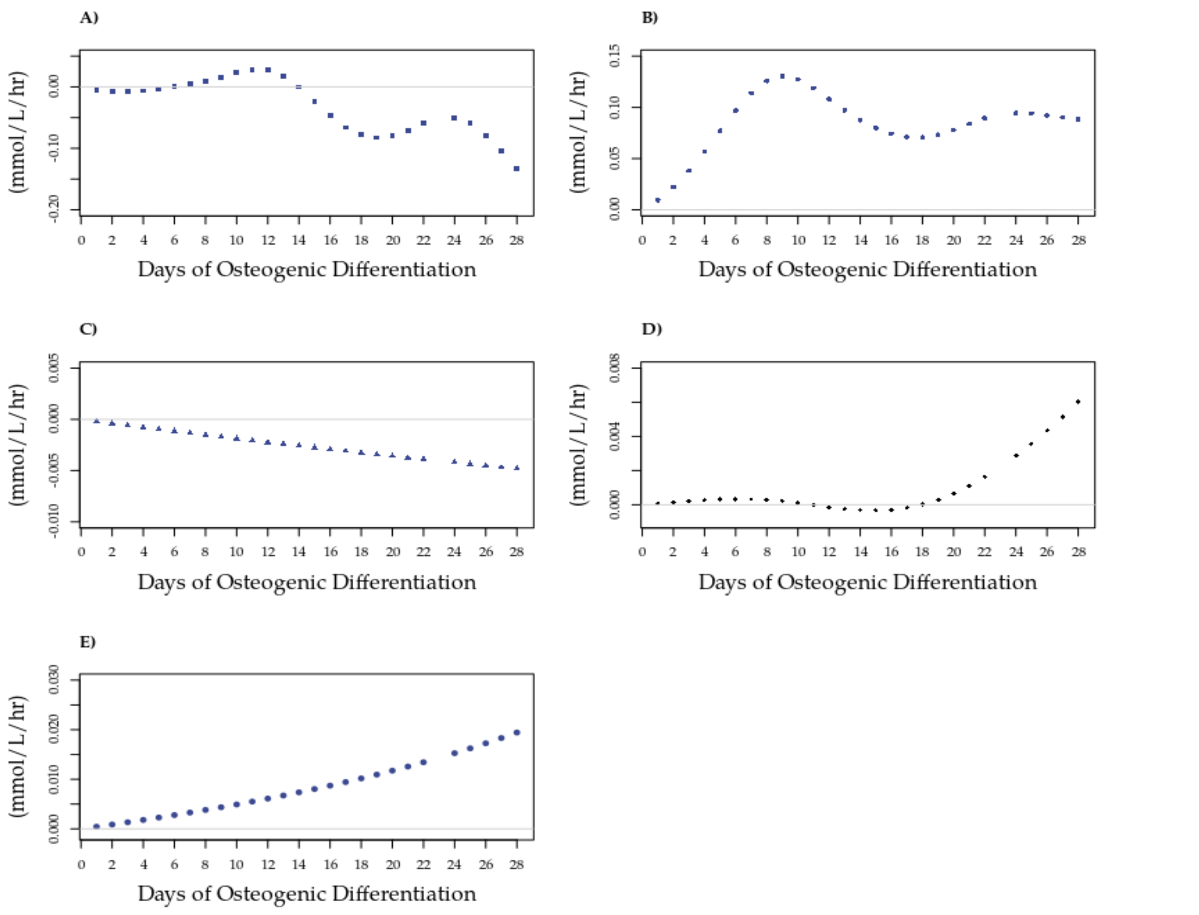

Figure S3. *Rate of concentration change (mmol/l/hr) in spent medium for extracellular (A) glucose (n = 4), (B) lactate (n = 4), (C) glutamine (n = 4), (D) ammonia (n = 4), and (E) glutamate (n = 4) for Days 1 to 28 of osteogenic differentiation. Dots (various shapes) represent the model predicted value subsequently used in change point analysis.*

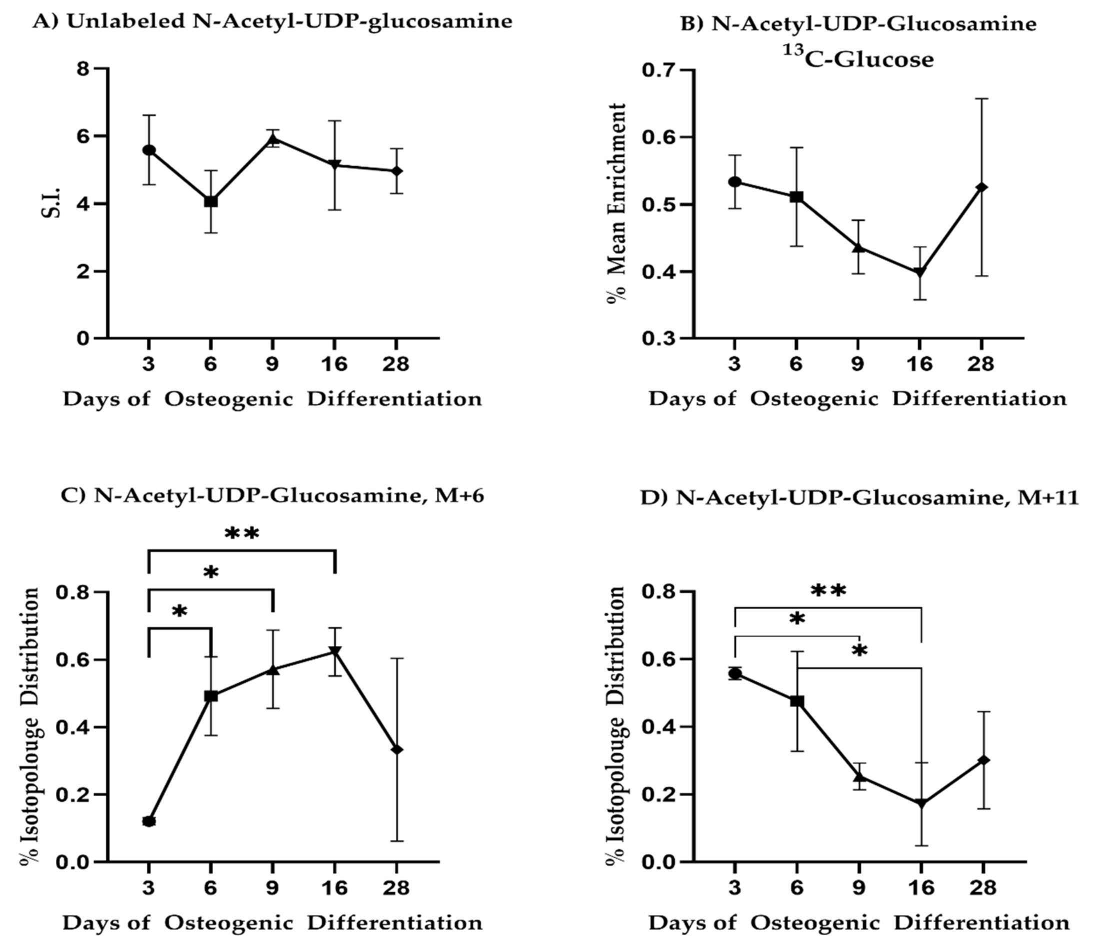

Figure S4. Isotopologue enrichment of N-Acetyl-UDP-Glucosamine is consistant with incresed glycolysis but decreased PPP activity*. A) Absolute Concentration of N-Acetyl-UDP-Glucosamine in unlabelled intracellular data (n = 3, SD), B) Mean ^{13}C-glucose label enrichment (n = 3, SD) C) Average % isotopolouge distribution of the M+6 isotopolouge for N-Acetyl-UDP-Glucosamine (n = 3, SD). Significant difference indicated with *or** D) Average % isotopolouge distribution of the M+11 isotopolouge for N-Acetyl-UDP-Glucosamine (n = 3, SS). * P<0.05 or **P <0.0099. S.I = Signal intensity.*

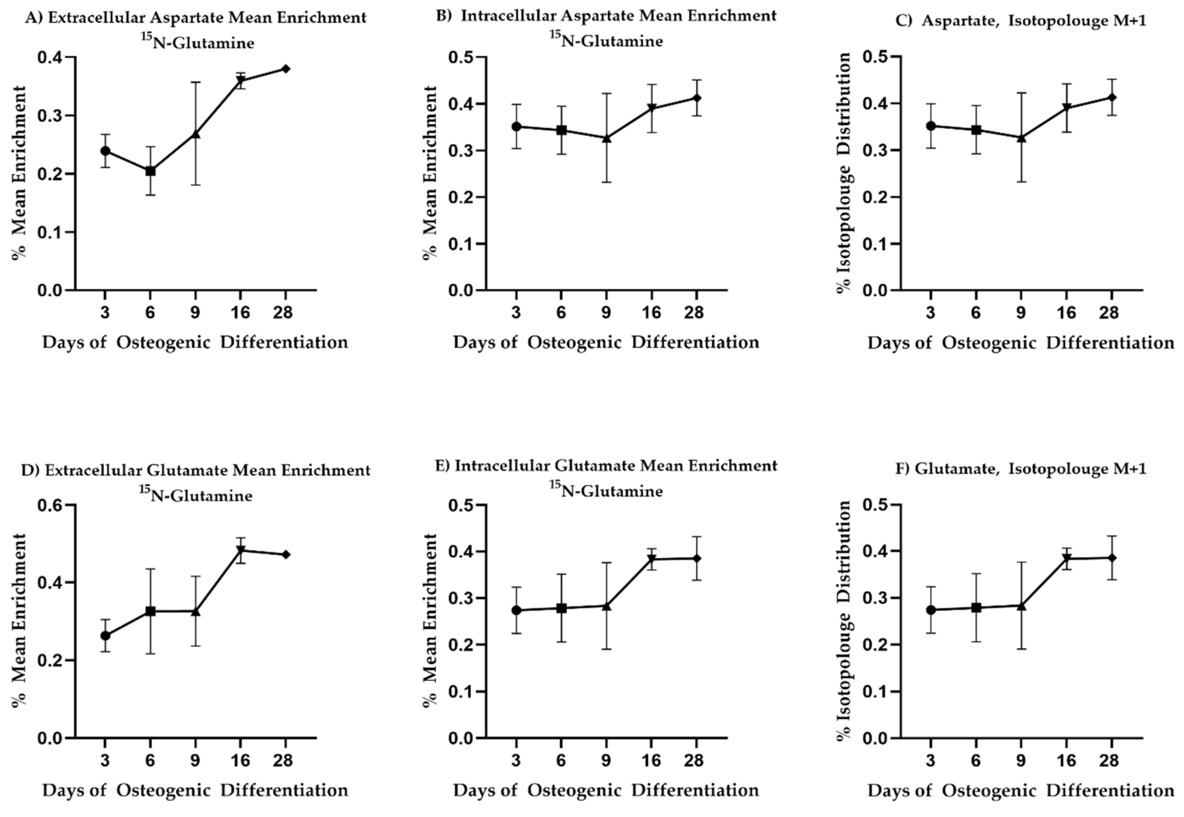

Figure S5. ¹⁵N- glutamine label incorporation and isotopologue enrichment of aspartate and glutamate. *A) Extracellular mean label enrichment of aspartate (n = 3, SD), B) Intracellular mean label enrichment of aspartate (n = 3, SD) C) Average % isotopolouge distribution of the M+1 isotopolouge for aspartate (n = 3, SD) D) Extracellular mean label enrichment of glutamate (n = 3, SD), E) Intracellular mean label enrichment of glutamate (n = 3, SD) (n = 3, SD) F) Average % isotopolouge distribution of the M+1 isotopolouge for glutamate (n = 3, SD)*

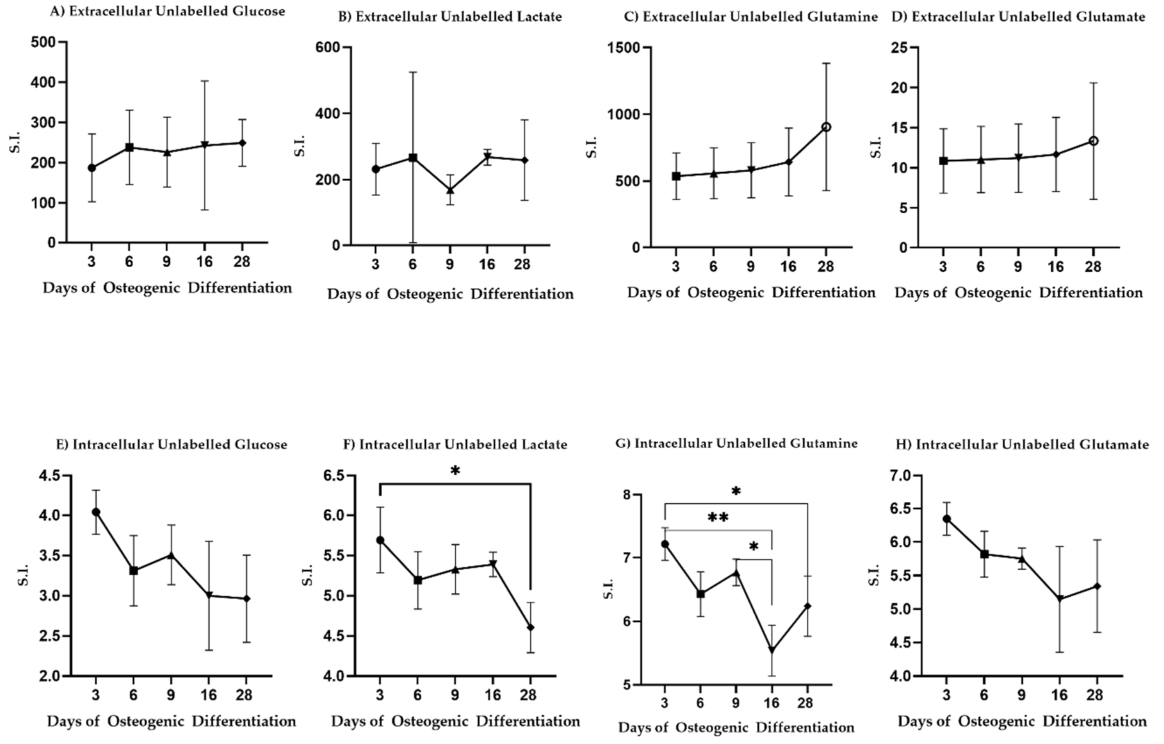

Figure S6. Intracellular and extracellular absolute concentration levels for A) and E) glucose, B) and F) lactate, C) and G) glutamine, and D) and H) glutamate (n = 3, SD in all instances). *S.I = signal intensity * P<0.05 or **P <0.0099.*
